# Luminescent Perhalofluoro Trityl Radicals

**DOI:** 10.1021/jacs.5c16418

**Published:** 2025-11-10

**Authors:** Johanna Schlögl, Alexander R. Krappe, Paul C. Fürstenwerth, Amelie L. Brosius, Carlo Fasting, Kurt F. Hoffmann, Ute Resch-Genger, Siegfried Eigler, Simon Steinhauer, Sebastian Riedel

**Affiliations:** † Institut für Chemie und Biochemie – Anorganische Chemie, 9166 Freie Universität Berlin, Fabeckstraße 34/36, 14195 Berlin, Germany; ‡ Institut für Chemie und Biochemie (SupraFAB), 54203 Freie Universität Berlin, Altensteinstraße 23a, 14195 Berlin, Germany; § Department 1, Division Biophotonics, 42220 Bundesanstalt für Materialforschung-und-prüfung (BAM), Richard-Willstätter Straße 11, 12489 Berlin, Germany

## Abstract

In this proof-of-concept study, we show that polyfluorinated trityl radicals with the, to this date, highest fluorination grade can be accessed in quantitative yields in a straightforward manner starting from the perfluorinated trityl cation. The trityl skeleton is functionalized with trimethylsilyl halides to yield perhalofluoro trityl cations, which are subsequently reduced using commercial zinc powder. In this way, we prepare three perhalofluoro trityl radicals and analyze the impact of the fluorine ligands on their electro-optical properties, revealing some interesting trends. In comparison to literature-known polychlorinated trityl radicals, the new polyfluorinated derivatives exhibit substantially higher fluorescence quantum yields, longer luminescence lifetimes, and an expanded emission range that extends into the yellow spectral region. They further display enhanced photostability under light irradiation. In radical-stained polystyrene nanoparticles, an additional broad emission band in the red–NIR wavelength region is observed, which is attributed to excimer formation. Finally, the stability of the new radicals is investigated under ambient conditions, showing the slow conversion with atmospheric oxygen yielding the respective peroxides, which are characterized by single-crystal X-ray diffraction. All in all, our study extends the present scope of luminescent trityl radicals, as the functionalization of the perfluorinated cationic precursor unlocks the path toward a vast variety of polyfluorinated trityl radicals.

## Graphical abstract

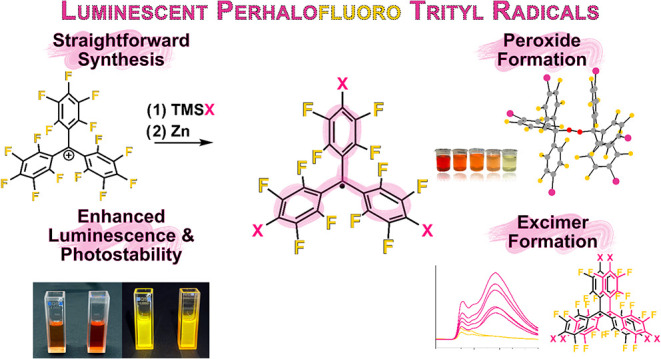

## Introduction

Fluorinated triphenylmethyl (trityl) cations have attracted a lot of attention lately, due to their superelectrophilic nature, that enables exceptional reactivities, such as the activation of short-chain alkanes.^1–5^ As reported recently by our group, the perfluorinated trityl cation [C­(C_6_F_5_)_3_]^+^ and its *para*-halogenated analogues [C­(C_6_F_4_Cl)_3_]^+^ and [C­(C_6_F_4_Br)_3_]^+^ are quantitatively accessible using commercially available GaCl_3_ and can be handled at room temperature for at least 1 day.^5^ Still, at ambient temperature, traces of radical species can be found. This intrigued us to turn our attention toward perhalofluoro trityl radicals.

Trityl radicals have a long history of 125 years. In 1900, Gomberg prepared its first representative, C­(C_6_H_5_)_3_, a discovery that marked the beginning of a new era in chemistry—of persistent organic radicals (Figure 1).^6^ However, Gomberg’s system suffered from low thermodynamic stability, as the central carbon atom reacts with the *para*-carbon atom of another equivalent in solution to form the so-called “Gomberg dimer” (Figure 1).^7–10^ This problem was later solved by Ballester, who introduced the per- and polychlorinated trityl radicals C­(C_6_Cl_5_)_3_ (PTM) and C(C_6_Cl_3_H_2_)_3_ (TTM) (Figure 1).^11^ These species do not dimerize in solution, which is attributed to the sterically protected chlorinated *para*-position. Furthermore, the central carbon atom is shielded by the *ortho*-chlorine ligands, which renders these radicals practically inert.^11^ Since then, the number of reports on polychlorinated trityl radicals has increased immensely, gaining a lot of recognition for their electrical, optical, and magnetic properties, due to their open-shell nature.^12–19^

**Figure 1:**
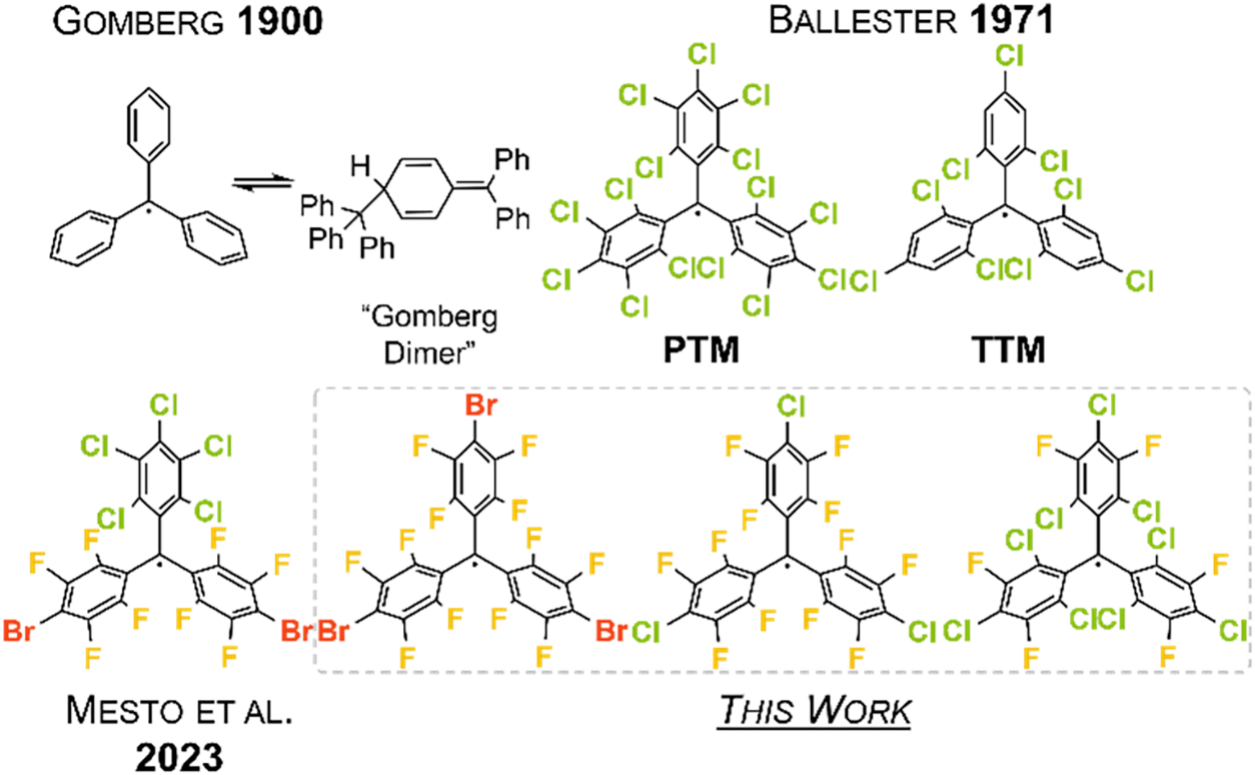
Top: Gomberg’s radical, its “dimer” and per- and polychlorinated trityl radicals by Ballester.^6–9,11^ Bottom: The only perhalofluoro trityl radical currently known and the radicals presented in this work.^15^

A main field of interest is the investigation of their luminescence and their application as so-called doublet emitters that may outperform classic organic closed-shell emitters.^12,13^ The trityl framework can easily be functionalized in order to tune certain properties, such as fluorescence quantum yields, lifetimes, or emission maxima.^13^ In contrast to their chlorinated counterparts, the impact of fluorine on the optoelectronic properties of trityl radicals has not been studied to the same extent, due to the scarcity of fluorinated representatives. In 1966, Filler and co-workers reported an EPR study of the perfluorinated trityl radical.^20–23^ Through dissolution of perfluoro trityl methanol in concentrated sulfuric acid and subsequent reduction of the so-formed cation with TiCl_3_, they obtained a white powder with “varying amounts of the radical”.^20^ However, the synthetic procedure is not described in detail.

The only other polyfluorinated trityl radical currently known is C­(C_6_Cl_5_)­(C_6_F_4_Br)_2_ (Figure 1).^15^ It is synthesized from the corresponding trityl methane, which is formed by a sequence of Friedel–Crafts reactions at high temperatures with an excess of the corresponding benzene building blocks present. Afterward, the trityl methane is deprotonated, and the anion is oxidized to give the radical. This synthetic strategy, however, has certain limitations when applied to the preparation of a broader range of polyfluorinated trityl radicals, as fluorinated benzene building blocks are not readily available and the use of such reactive species leads to synthetic challenges like side reactions and lower yields.^15^

Herein, we present an alternative route to access perhalofluoro trityl radicals, starting from the perfluorinated trityl cation, which can be readily functionalized using trimethylsilyl reagents.^5^ Afterward, the cationic species is reduced with commercial zinc powder, and the radicals can be isolated in nearly quantitative yields. In this way, three perhalofluoro trityl radicals are prepared and their electro-optical properties are analyzed, revealing some extraordinary trends. Finally, their reactivity toward oxygen is investigated that leads to the formation of the respective perhalofluoro trityl peroxides.

## Results and Discussion

As recently reported by our group, it is possible to halodefluorinate the perfluorinated trityl cation **15F** ^**+**^ in the *para*-positions using trimethylsilyl halides TMSX (X = Cl, Br) (Scheme 1).^5^ This gives trityl cations ***p*** **-3X12F** ^**+**^, which remain superelectrophilic like their parent perfluorinated analogue but are more thermally stable. The corresponding trityl radicals **3X12F^•^** can be obtained by adding zinc powder to a solution of ***p*** **-3X12F** ^**+**^ in SO_2_ClF at −30 °C, warming the reaction mixture to room temperature and stirring it for 12 h (Scheme 1). During that time, the suspension changes its color from violet (***p*** **-3Cl12F** ^**+**^) or blue (***p*** **-3Br12F** ^**+**^) to red and becomes fluorescent. After workup, the perhalofluoro trityl radicals **3Cl12F^•^** and **3Br12F^•^** can be isolated, respectively, as orange-red and dark red powders. The reaction can also be performed in *ortho*-difluorobenzene; the yield is only slightly lower, due to side reactions occurring between the cationic species ***p*** **-3X12F** ^**+**^ and the solvent (see Supporting Information for more information).

**Scheme 1:**
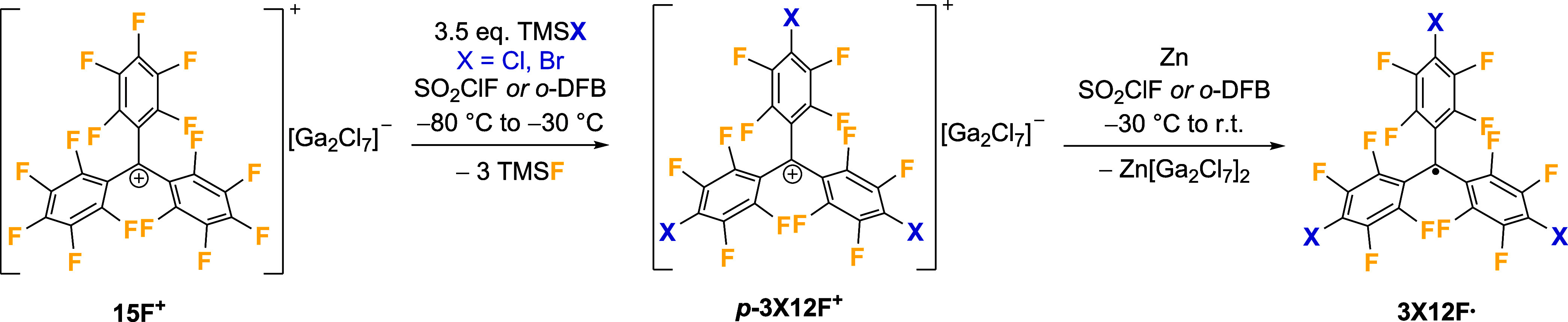
Synthesis of 3X12F^•^ via Reduction of the Cationic Species *p*-3X12F^+^ Using Zinc Powder (X = Cl, Br)

As also reported previously, leaving a solution of ***p*** **-3Cl12F** ^**+**^ for a month at room temperature leads to the formation of another cationic trityl species **9Cl6F** ^**+**^.^5^ This happens most likely due to a side reaction of ***p*** **-3Cl12F** ^**+**^ with its chloro-gallate counteranion leading to a second chlorodefluorination in the *ortho*-positions. Inspired by this reactivity, an excess of trimethylsilyl chloride was added to **15F** ^**+**^ in SO_2_ClF, and the reaction mixture was stirred at room temperature for 5–7 days (Scheme 2). The stepwise chlorodefluorination can be observed by a color change from berry-red to violet within the first 10 min corresponding to the formation of ***p*** **-3Cl12F** ^**+**^, followed by a second color change to blue after a few days, attributed to the formation of **9Cl6F** ^**+**^. Analysis of the reaction mixture by low-temperature ^19^F NMR spectroscopy reveals one main signal at −140.4 ppm that can be ascribed to the *meta*-fluorine atoms of **9Cl6F** ^**+**^ (Figure S9). Besides that, however, there are some unidentified side products probably resulting from the long reaction time and the highly reactive mixture. The UV–vis spectrum displays an absorption maximum at 638 nm (Figure S11). The bathochromic shift going from **15F** ^**+**^ to ***p*** **-3Cl12F** ^**+**^ to **9Cl6F** ^**+**^ correlates with a decreasing HOMO–LUMO gap (Figure S12 and Table S1). After the addition of zinc to a solution of **9Cl6F** ^**+**^ in SO_2_ClF, the color changes from blue to dark red and the suspension turns fluorescent (Scheme 2). After workup, **9Cl6F^•^** is obtained as a deep red powder. Please note that this reaction cannot be performed in *ortho*-difluorobenzene. Furthermore, it is not possible to access the corresponding *ortho*- and *para*-brominated species 9Br6F^+^ and 9Br6F^•^ by using an excess of trimethylsilyl bromide, as the latter is oxidized in the presence of ***p*** **-3Br12F** ^**+**^ to yield elemental bromine.

**Scheme 2:**
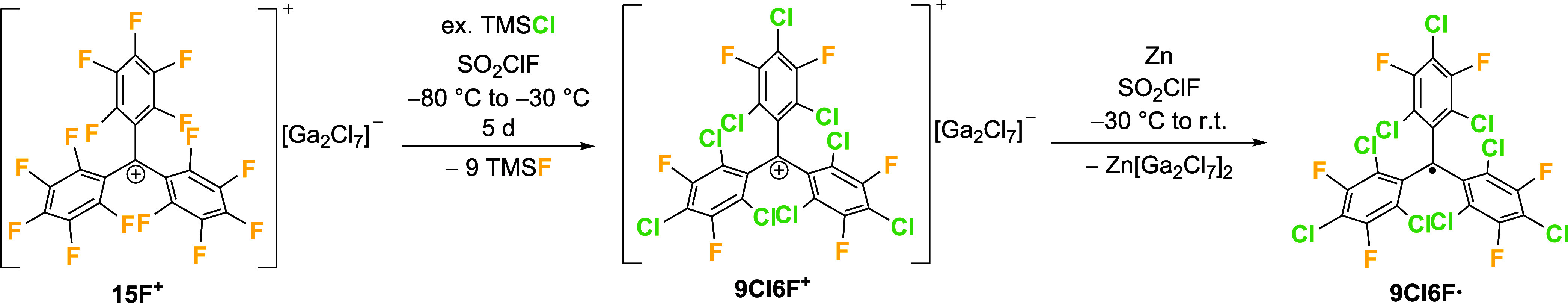
Synthesis of 9Cl6F^+^ via Chlorodefluorination of the Perfluorinated Analogue 15F^+^ Using an Excess of TMSCl Followed by Reduction Using Zinc Powder Leading to 9Cl6F^•^

Single-crystal X-ray diffraction reveals a planar environment around the *sp* ^2^-hybridized central carbon atom and a propeller-like arrangement of the phenyl rings for all three perhalofluoro trityl radicals (Figure 2). The sum of the angles around the carbon center is 360° in all three cases. A significant difference lies in the twisting angles of two phenyl moieties around the central carbon atom: the larger van-der-Waals radii of the *ortho*-chlorine atoms of **9Cl6F^•^** compared to the *ortho*-fluorine atoms of **3X12F^•^** result in a larger steric congestion around the central carbon atom and by that an increased dihedral angle φ (Figure 2d). The dihedral angles range from 26.6° to 45.5° for **3Cl12F^•^**, 34.4° to 47.2° for **3Br12F^•^**, and 42.2° to 52.4° for **9Cl6F^•^**.

**Figure 2:**
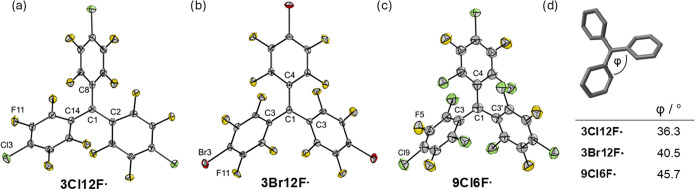
Molecular structures of all three perhalofluoro trityl radicals in the solid state. Thermal ellipsoids are set at 50% probability. (a) **3Cl12F^•^**. Selected bond lengths (pm) and angles [deg]: C1–C14 146.7(6), C1–C2 144.5(4), C1–C8 146.3(5), C14–C1–C8 118.6(3), C2–C1–C8 121.9(3), C14–C1–C2 119.5(3) (b) **3Br12F^•^**. Selected bond lengths [pm] and angles [deg]: C1–C3 145.6(3), C1–C4 145.9(6), C3–C1–C4 119.0(2), C3′–C1-C4 119.0(2), C3–C1–C3′ 122.0(4) (c) **9Cl6F^•^**. Selected bond lengths [pm] and angles [deg]: C3–C1 145.9(1), C1–C4 146.6, C3–C1–C4 120.3(6), and C3–C1–C3′ 119.4(4). (d) Experimental dihedral angles φ of **3X12F^•^** and **9Cl6F^•^**.

Isotropic X-band EPR spectra of all three trityl radicals in toluene at 298 K show no resolved hyperfine splitting and rather broad line widths (Figure 3a). This is attributed to the large number of magnetic nuclei interacting with the unpaired electron.^24,25^ Furthermore, spin–orbit coupling resulting from the presence of halide ligands and quadrupolar effects from the bromide ligand may further contribute to the line broadening.^25^ The narrower line width of **9Cl6F^•^** compared to **3X12F^•^** likely results from a stronger twisting around the central carbon atom due to the *ortho*-chlorine ligands, weakening the π-conjugation over the trityl framework (Figures 2d and 3b). The isotropic *g*-values fit those calculated on the B3LYP/def2-SVP level of theory (Table 1).^26–29^ The increase of the g-factor going from the chlorinated to the brominated species is explained by stronger spin–orbit interactions, which was already observed for similar systems.^16^ Interestingly, additional chlorine ligands in the *ortho*-positions do not affect the *g*-value, as the same *g*_iso_ is observed for both **3Cl12F^•^** and **9Cl6F^•^** (Table 1).

**Figure 3:**
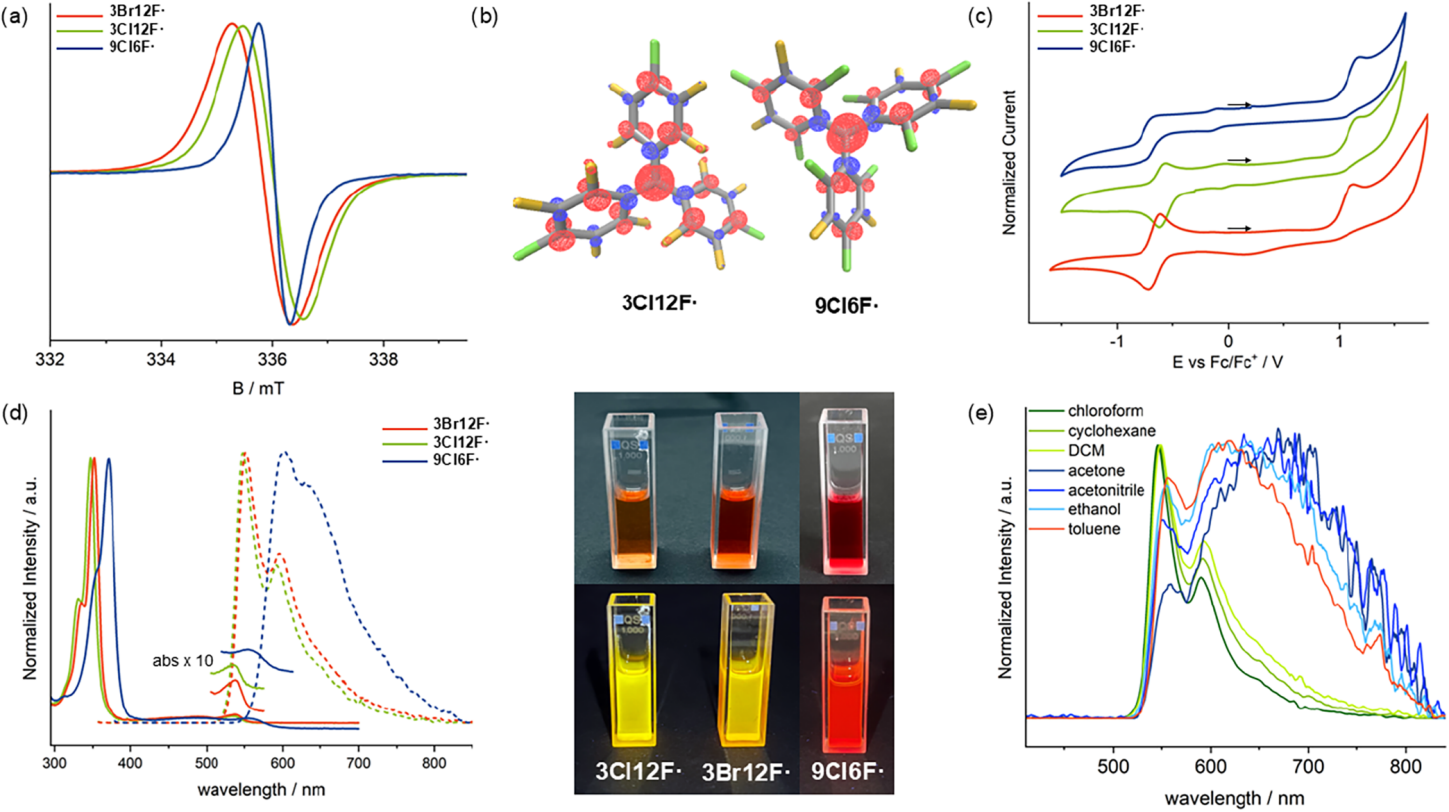
(a) Isotropic X-band EPR spectra of **3X12F^•^** and **9Cl6F^•^** in toluene at 298 K. (b) Calculated spin density of **3Cl12F^•^** and **9Cl6F^•^** at the B3LYP-D3­(BJ)/def2-TZVPP level of theory (iso = ±0.005).^26–29^ (c) Cyclic voltammograms of **3X12F^•^** and **9Cl6F^•^** in CH_2_Cl_2_ at 298 K (0.1 M NBu_4_PF_6_, 100 mV s^–1^). (d) Normalized UV–vis absorption (solid lines) and fluorescence emission (dashed lines) spectra of **3X12F^•^** and **9Cl6F^•^** in deaerated CHCl_3_ at 298 K. The fluorescence emission spectra were excited at 350 nm (**3X12F^•^**) and 370 nm (**9Cl6F^•^**). (e) Normalized fluorescence spectra of **3Cl12F^•^** in various deaerated organic solvents at 298 K. All spectra have been normalized at the respective absorption maximum.

**Table 1:** Magnetic, Electrochemical, and Optical Properties of the Herein Presented Perhalofluoro Trityl Radicals in Comparison with Their Chlorinated Analogues

	*g*_iso_ ^a^	λ_abs_, nm^b^	λ_em_, nm^b^	Φ, %^b^	τ, ns^b^	*E*_1/2_^an/rad^, V^c^	*E*_ox_^rad/cat^, V^c^
PTM^13,37,38^	2.003	385	605	1.6	7.0		
TTM^35,36^		370	570	2.0	7.0		
**3Cl12F^•^**	2.004 (2.004)	347, 330 (sh)	547, 590 (sh)	9.0 (5.6)	20.0 (17.0)	–0.595	1.163
**3Br12F^•^**	2.006 (2.007)	350, 330 (sh)	549, 595 (sh)	8.4 (5.9)	27.5 (19.8)	–0.663	1.119
**9Cl6F^•^**	2.004 (2.004)	354 (sh), 370	587, 628 (sh)	3.6 (6.8)	19.0 (30.4)	–0.743	1.174

a Experimental *g*_iso_ values. In brackets are the calculated values obtained at the B3LYP-D3­(BJ)/def2-SVP level of theory.^26–29^

b Absorption and emission spectra measured in deaerated CHCl_3_ (sh = shoulder). Absolute PLQY Φ and lifetimes τ measured in deaerated C_6_H_12_ and CHCl_3_ (values given in brackets) at 298 K.

c Redox potentials determined in CH_2_Cl_2_ at 298 K (0.1 M NBu_4_PF_6_, 100 mV s^–1^). Redox potentials are referenced to Fc/Fc^+^.

The cyclic voltammograms of the three trityl radicals reveal two redox processes: a quasi-reversible one corresponding to the anion-radical redox couple and an irreversible process attributed to the oxidation of the radical to the cation (Figure 3c). The irreversibility is explained by the previously described superelectrophilicity of the cationic species, which probably results in a hydride or chloride ion abstraction from the solvent.^5^

The UV–vis spectra measured in deaerated chloroform at 298 K reveal an absorption pattern typical for trityl radicals comprising an intense absorption band below 400 nm, a lower band overlapping with the former, and a weak absorption in the visible region (Figure 3d).^30^ In comparison to the perchlorinated trityl radical 15Cl**^•^**, the presence of fluorine ligands induces a pronounced blue shift that increases with the number of fluorine ligands (Table 1). The same can be seen in the fluorescence emission spectra that were also recorded in chloroform at 298 K (Figure 3d and Table 1). Note that the radicals **3Cl12F^•^** and **3Br12F^•^** show the most blue-shifted emission among all known trityl radicals.^13,15^ The type of halide ligand in the *para*-position does not seem to have a significant impact on the position of the absorption or emission bands, as can be seen by the almost negligible bathochromic shift when comparing the *para*-chlorinated and *para*-brominated radicals (Table 1).

The electronic transitions of all three compounds are comparable according to time-dependent density functional theory (TD-DFT) (Figure S34 and Table S4). The intense absorption bands at about 350 nm originate from higher electronic excitations, namely, D_0_ → D_7_ and D_0_ → D_8_ transitions, in which primarily the unpaired electron from the SOMOα is excited to LUMOα and LUMO + 1α, so-called α-excitations.

In contrast, the weak and unstructured absorption in the visible region is attributed to β-excitations from lower orbitals to the SUMOβ orbital. At first, the compounds therefore appear to have a large Stokes shift because the transitions D_0_ → D_1_ up to D_6_ are barely visible due to low oscillator strengths and vibrational broadening, as determined by excited-state dynamic calculations (Figure S35). Nevertheless, the emission proceeds according to Kasha’s rule from the energetically lowest excited state D_1_. Overall, these observations are in accordance with the electronic structural model for alternant π-hydrocarbon radicals.^31,32^

The absolute photoluminescence quantum yields and fluorescence lifetimes were measured in cyclohexane and chloroform at 298 K (Table 1). The obtained values are rather low, which, however, is common for these types of radicals.^12,13^ To improve their luminescence properties, further tailoring with functional groups like pyridyl or carbazolyl units is usually required.^13,33–35^ Nevertheless, the quantum yields of the polyfluorinated trityl radicals **3Cl12F^•^**, **3Br12F^•^**, and **9Cl6F^•^** significantly exceed those of PTM by 225–560% (Table 1). This trend is reflected by the respective luminescence lifetimes, which are increased by a factor of 3–4 compared to PTM. A similar enhancement of the photoluminescence properties upon exchanging chlorine for fluorine substituents was previously reported for diphenylpyridylmethyl radicals.^39–41^

The latter also shows significantly improved photostabilities in comparison with the benchmark radical TTM.^35^ Herein, the photostability of **3Cl12F^•^** was exemplarily examined. A solution of **3Cl12F^•^** in deaerated CHCl_3_ (0.1 mM) was irradiated at 350 nm, yielding a half-life *t*_1/2_ of 7974 s, which exceeds the one measured for TTM by a factor of about 25 under comparable conditions (Figure S30).^35^ This remarkable improvement in photostability may be attributed to the *ortho*-fluorine substituents, which are less susceptible to photocyclization reactions than the corresponding *ortho*-chlorine substituents.^42^

These results show that with the herein presented perhalofluoro trityl radicals, significantly higher quantum yields and luminescence lifetimes are possible without the need for additional functional group tailoring. Furthermore, highly fluorinated radicals, such as **3Cl12F^•^** and **3Br12F^•^**, extend the overall fluorescence emission range of trityl radicals into the yellow region (Figure 3d). In combination with even stronger electron-withdrawing groups in the *para*-position, fluorescence in the green region may now be accessible.

The perhalofluoro trityl radicals **3X12F^•^** exhibit closely matching and similarly shaped UV–vis absorption spectra in chloroform, cyclohexane, methylene chloride, acetone, acetonitrile, ethanol, and toluene (Figures S24 and S25 and Table S2). In contrast, the fluorescence emission behavior of these radicals is strongly affected by the choice of the solvent. In chloroform, cyclohexane, and methylene chloride closely matching and similarly shaped emission spectra with vibronic progression are observed (Figures 3e and S28 and Table S2), which is indicative of a locally excited (LE) state emission.^43^ In polar or aromatic solvents, such as acetone, acetonitrile, ethanol, and toluene, a red-shifted, broad emission band of significantly lower intensity appears, with its spectral position depending on the solvent polarity (Figures 3e and S28 and Table S2). The broad shape is indicative of strong solute–solvent interactions. Moreover, the radical fluorescence is significantly quenched in aromatic or polar solvents, as reflected by the photoluminescence quantum yields dropping to values below 1% (Table S3). Although this effect is not fully understood, we propose the involvement of a charge-transfer (CT) excited state, stabilized either by polar solvent molecules due to electrostatic effects or by aromatic solvent molecules through π–π interactions, favoring nonradiative depopulation.^43^ TD-DFT calculations reveal a strong increase in polarity of the D_1_ state of roughly 10 D in contrast to the nonpolar D_0_ state, further supporting this assumption. The dual emission of **3X12F^•^** in polar or aromatic solvents could originate, e.g., from emission of the radicals’ initially relaxed excited state with a relatively planar structure (LE state) and from a more polar excited state with a more pronounced CT character.^44^

The *para*-chlorinated trityl radical **3Cl12F^•^** can be readily incorporated into polystyrene nanoparticles (PS-NPs), showing unaltered emission spectra at low concentrations with an emission maximum at 547 nm. At higher radical loading concentrations, however, an additional broad, structureless emission band emerges at longer wavelengths (Figure 4). The intensity of this emission band, located at 685 nm, increases proportionally with the radical concentration (Figure 4). A similar behavior was previously reported for the TTM radical incorporated in organic nanoparticles and polymeric films and for the (3,5-dichloro-4-pyridyl)­bis­(2,4,6-trichlorophenyl)­methyl radical (PyBTM) embedded in a host crystal of its hydrogenated closed-shell precursor (PyBTM-H).^45,46^ The red-shifted, broad emission of these radicals was attributed to the formation of a “radical-excimer,” a transient complex formed between an excited-state and a ground-state trityl radical within the respective matrix, which may also account for the red-shifted emission of **3Cl12F^•^** in PS-NPs.^46^ The excimer is assumed to be formed between molecules that are already in close proximity prior to excitation.^47^

**Figure 4:**
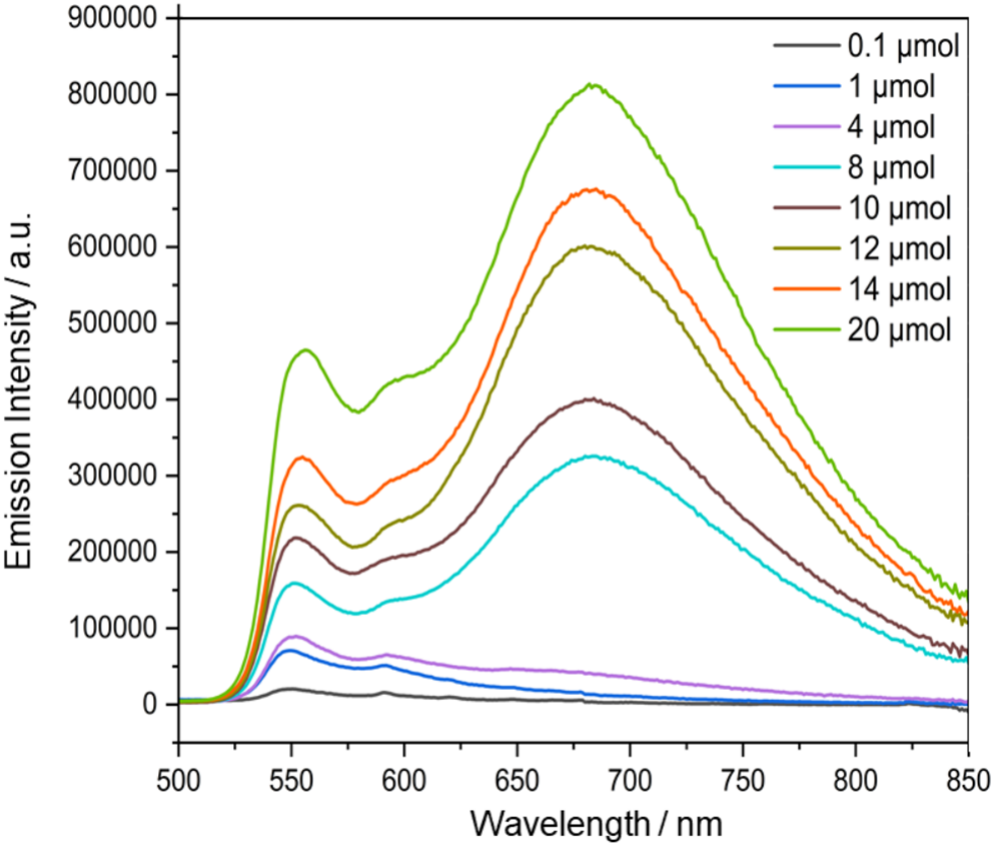
Emission spectra of **3Cl12F^•^** in 200 nm PS-NPs with an increasing loading concentration.

As described earlier, polychlorinated trityl radicals are characterized by their extraordinary thermodynamic stability and chemical inertness. This is traced back to the chlorine substituents in the *ortho*- and *para*-positions, which effectively prevent dimerization and shield the radical’s center from attack by small molecules.^11^ In the same way, the perhalofluoro trityl radical **9Cl6F^•^** also exhibits remarkable inertness. For trityl radicals, **3X12F^•^** dimerization is likewise suppressed since their *para*-position is protected by the halide substituent. However, in this case, the central carbon atom is less sterically shielded, due to the smaller van-der-Waals radii of the *ortho*-fluorine substituents compared to their chlorinated counterparts, as evident from the space-filling model (Figure 5a).

**Figure 5:**
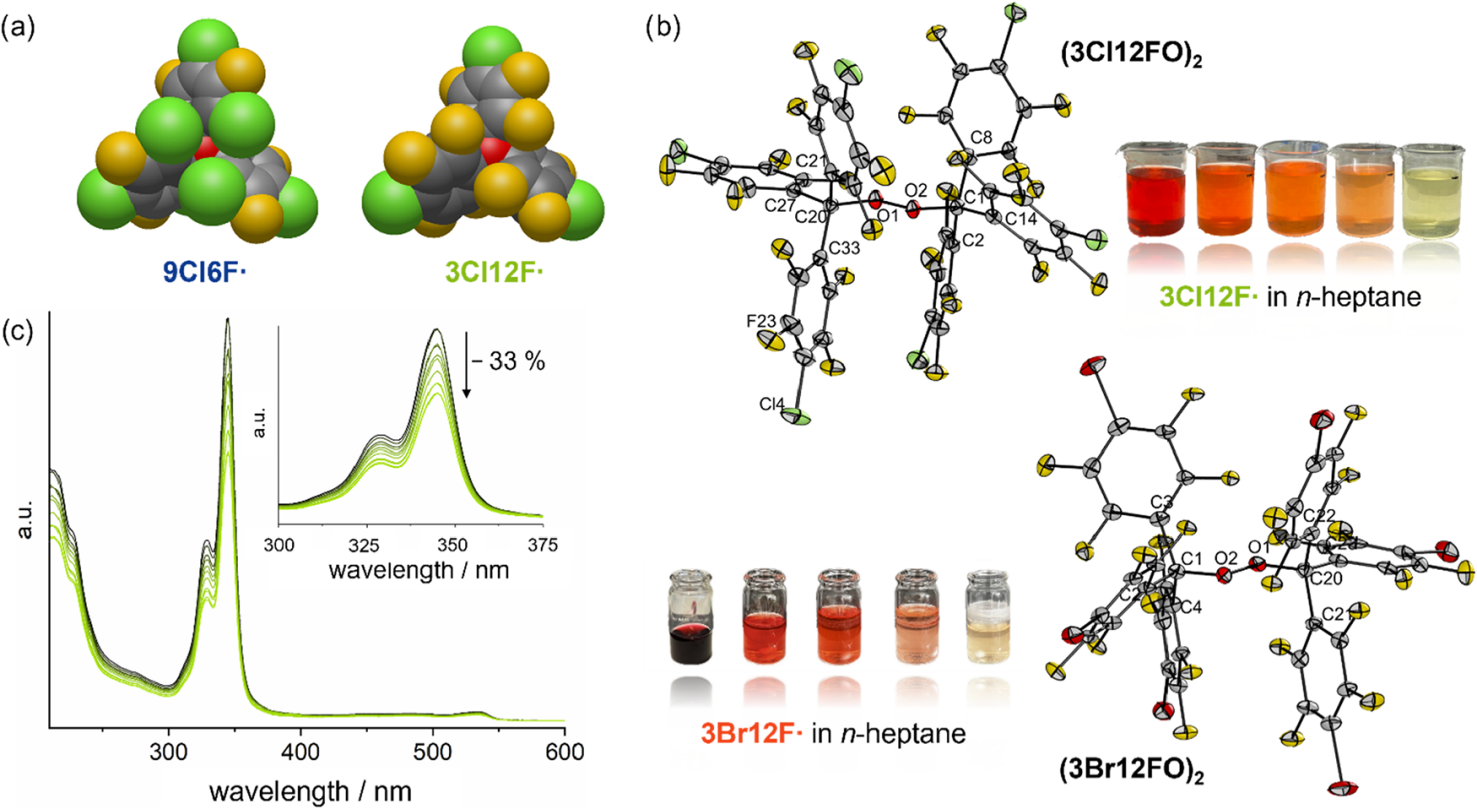
(a) Space-filling model of **3Cl12F^•^** and **9Cl6F^•^** (B3LYP-D3­(BJ)/def2-TZVPP). The central carbon atom is marked in red. (b) Molecular structures of both perhalofluoro trityl peroxides in the solid state. Thermal ellipsoids set at 50% probability **(3Cl12F-O)**_**2**_. Selected bond lengths [pm] and angles [deg]: C1–C2 153.6(4), C1–C14 155.1(4), C1–C8 154.3(4), C1–O2 144.5(3), O1–O2 148.3(3), C2–C1–C14 115.9(2), C14–C1–C8 104.9(2), C8–C1–O2 113.6(2), O2–C1–C2 105.6(2), C1–O2–O1-C20 175.7(2). **(3Br12F-O)**_**2**_. Selected bond lengths [pm] and angles [deg]: C1–C2 155.7(1), C1–C3 154.5(1), C1–C4 154.9(1), C1–O2 143.8(1), O1–O2 147.8(1), C3–C1–C2 104.9(4), C2–C1–C4 114.7(5), C4–C1–O2 108.0(6), O2–C1–C3 114.4(5), and C1–O2–O1-C20 178.0(5). Photographs show the discoloration of the *n*-heptane solutions of both **3X12F^•^** over the course of 1 week. (c) Time-resolved UV–vis absorption spectra of **3Cl12F^•^** in *n*-heptane, recorded over 7 days to follow its reaction with atmospheric oxygen (see Supporting Information for more information).

Thus, when a solution of **3X12F^•^** in *n*-heptane is left at ambient conditions for 7 days (with evaporated *n*-heptane continuously replenished), a gradual discoloration of the red solution as well as the precipitation of colorless crystals is observed (Figure 5b). The ^19^F NMR spectrum of each reaction mixture reveals two signals that can be assigned, respectively, to the *ortho*- and *meta*-fluorine atoms at −135.7 and −140.5 ppm for **(3Cl12F**–**O)**_**2**_ and −133.1 and −140.7 ppm for **(3Br12F**–**O)**_**2**_ (Figures S16 and S19). Both ^13^C­{^19^F} NMR spectra exhibit a signal around 84 ppm, which is characteristic for the quaternary carbon atom bound to the peroxide unit (Figures S17 and S20).^48^ Please note that minor additional signals in the ^19^F NMR spectra indicate small amounts of impurities; the reaction conditions were not optimized, as the goal of this study was solely to probe the reactivity of the radicals rather than to establish a synthetic route to trityl peroxides.

Single-crystal X-ray diffraction analysis reveals the molecular structure of the respective peroxide **(3X12F-O)**_**2**_ in the solid state (Figure 5b). The O–O bond distance for both peroxides is 148 pm, which is the same value as for the Gomberg trityl peroxide and in the same range as the perfluoro-*tert*-butyl peroxide.^49,50^ The COOC dihedral angle is 176° for **(3Cl12F-O)**_**2**_ and 178° for **(3Br12F-O)**_**2**_, which is far from the ideal peroxide dihedral angle of 120°. This deviation has already been observed for other peroxides and can be rationalized by different factors, such as steric repulsion, electron-pair repulsion, and orbital interactions.^50,51^ The dihedral angles of the herein presented peroxides lie in between those of the nonfluorinated and the perfluorinated *tert*-butyl peroxides.^50,52^

All in all, trityl radicals **3X12F^•^** are persistent rather than inert: they do not undergo dimerization but react with oxygen to form the corresponding peroxides. To assess the practical implications of this reactivity under ambient conditions, we performed time-dependent UV–vis absorption measurements, exemplarily with an air-saturated solution of **3Cl12F^•^** in *n*-heptane (Figure 5c, see Supporting Information for more information). The absorption spectra reveal a decrease by about 8% within the first 24 h and a reduction in absorption intensity by 33% after 7 days. This demonstrates that the reaction between the trityl radicals **3X12F^•^** and oxygen is relatively slow such that the radicals can be handled in solution under ambient conditions for at least a few hours without significant decomposition. This outcome is also consistent with our own experience working with **3X12F^•^** radicals.

## Conclusions

In this work, we presented a straightforward route to perhalofluoro trityl radicals starting from the respective perfluorinated trityl cation, which can be functionalized using trimethylsilyl halides and reduced with commercial zinc powder. Using this approach, three perhalofluoro trityl radicals with the, to date, highest fluorination grade are accessible in nearly quantitative yields. The *ortho*-chlorinated trityl radical **9Cl6F^•^** is highly inert, whereas the *ortho*-fluorinated radicals **3X12F^•^** (X = Cl, Br) react with oxygen to form the corresponding peroxides, which were characterized by single-crystal X-ray diffraction. However, the reaction with oxygen is slow, allowing these persistent radicals to be handled under ambient conditions without significant decomposition for at least a few hours. For the first time, it is possible to study these highly fluorinated trityl species with regard to their magnetic, electrochemical, and optical behavior. Remarkable electro-optical properties were found, including the highest photoluminescence quantum yields and the longest luminescence lifetimes reported for nonfunctionalized, mixed-halide trityl radicals reported so far, as well as the most blue-shifted fluorescence emission and enhanced photostability. In polar and aromatic solvents, dual emission is observed for **3X12F^•^** with an additional broad, red-shifted emission band and significantly quenched fluorescence. Finally, **3Cl12F^•^** was successfully incorporated into polystyrene nanoparticles, where it exhibits a concentration-dependent emission with a new broad, red-shifted band, indicative of “radical excimer” formation as previously reported for the TTM and the PyBTM radicals in different solid matrices.^45,46^

With this proof-of-concept study, the scope of luminescent trityl radicals is extended, as the functionalization of the perfluorinated cationic precursor unlocks the path toward a vast variety of polyfluorinated trityl radicals.

## Supplementary Material



## Notes

General information on experimental procedures, spectral data, crystallographic data, and quantum-chemical calculations (PDF)
