# Supplementary material for: Luminescent Perhalofluoro Trityl Radicals
Source: J Am Chem Soc. 2025 Nov 10;147(46):43105–12. doi: 10.1021/jacs.5c16418 (PMC12636010; doi:10.1021/jacs.5c16418)
Supplement: Supplementary file 1 [file ja5c16418_si_001.pdf]

Luminescent Perhalofluoro Trityl Radicals

Johanna Schlögl[a], Alexander R. Krappe[b], Paul C. Fürstenwerth, Amelie L. Brosius[a], Carlo Fasting[a], Kurt F. Hoffmann[a], Ute Resch-Genger[c], Siegfried Eigler[b], Simon Steinhauer[a], Sebastian Riedel[a]*

[a] J. Schlögl, A. L. Brosius, Dr. C. Fasting, Dr. K. F. Hoffmann, Dr. S. Steinhauer, Prof. Dr. S. Riedel
Freie Universität Berlin, Institut für Chemie und Biochemie—Anorganische Chemie
Fabeckstraße 34/36, 14195 Berlin (Germany)
E-mail: s.riedel@fu-berlin.de
[b] A. R. Krappe, Prof. S. Eigler
Freie Universität Berlin, Institut für Chemie und Biochemie (SupraFAB)
Altensteinstr. 23a, 14195 Berlin (Germany)
E-mail: siegfried.eigler@fu-berlin.de
[c] P. C. Fürstenwerth, Dr. U. Resch-Genger
Bundesanstalt für Materialforschung und -prüfung (BAM) Department 1, Division Biophotonics
Richard-Willstätter Straße 11, 12489 Berlin (Germany)
E-mail: ute.resch@bam.de

Supporting Information

Inhalt

1. General Information....3
Experimental Information....3
Spectroscopic Measurements....3
Optical Measurements....3
Loading of polytyrene nanoparticles with 3Cl12F·....4
Photostability Measurements....4
Crystallographic Information....5
Computational Details....5
2. Syntheses, structure-analytical, and spectroscopical data....6
2.1. $C(C_6F_5)_3OH$....6
2.2 $C(C_6F_5)_3Cl$....6
2.3. 3Cl12F·....6
2.4.Scale-up....10
2.5. 3Br12F·....10
2.6. [9Cl6F][Ga_2Cl_7]....13
2.7. 9Cl6F·....16
2.8. $(3Cl12FO)_2$....18
2.9. $(3Br12FO)_2$....20
2.10. Stability of Perhalofluoro Trityl Radicals....23
3. Optical Data....25
3.1. Luminescence properties of 3X12F· in various organic solvents....25
3.2. Photostability of 3Cl12F·....30
3.3. Luminescence properties of 3Cl12F· in polystyrene nanoparticles....32
4. TD-DFT....33
5. Crystal Data....34
6. Quantum-chemical Calculations....40
Citations....45

1. General Information

Experimental Information

All preparative work was carried out under an inert atmosphere using standard Schlenk techniques. Glassware was greased with Triboflon III. All solid materials were stored and handled inside a glove box with an atmosphere of dry argon (O_2 < 0.5 ppm, H_2O < 0.5 ppm). SO_2ClF was dried and stored on CaH_2. All other solvents were dried using a MBraun SPS-800 solvent system and stored on molecular sieve. Reagents were purchased from standard commercially suppliers and used as received. Zinc powder (99.9%, 325 mesh) was purchased from abcr, stored inside a glove box, and used as received. Preparative normal phase MPLC was performed on an ISCO-Teledyn CombiFlash Rf+ system with integrated variable wavelength detection in gradient mode. Solvents were n-hexane/acetone mixtures, starting with 100% n-hexane. Pre-packed NP-columns (spherical silica, 20-35µm, 60A, 20g, Agela Technologies) were purchased from Phenomenex (Aschaffenburg, Germany).

Spectroscopic Measurements

IR spectra were measured on a Bruker ALPHA FTIR spectrometer equipped with a diamond ATR attachment in a glove box filled with argon (resolution 4 cm^{-1}). Liquid NMR spectra were recorded on a JEOL 400 MHz ECS or ECZ spectrometer in J. Young NMR tubes. All reported chemical shifts were referenced to the Ξ values given in IUPAC recommendations of 2008 using the 2H signal of the deuterated solvent as internal standard or a capillary filled with acetone-d_6, trichlorofluoromethane, tetramethylphosphine oxide and tetramethylsilane as external standard (ext. acetone-d_6). EPR spectra were recorded on a X-band Magnetech MS 5000 spectrometer at 298 K. Samples were sealed in 50µl capillaries. Simulations were performed with Easyspin.[1] Raman spectra were recorded on a Bruker MultiRAM II equipped with a low-temperature Ge detector (1064 nm, 200 mW, resolution 2 cm^{-1}). Cyclic Voltammetry measurements were performed using a Bio Logic Science Instruments Pvt. Ltd. SAS model SP-300 potentiostat and the software EC-Lab® with a three-electrode set-up consisting of platinum wires. The measurements were performed at a scan rate of 100 mV/s in anhydrous solvents under argon atmosphere. The voltammograms were internally referenced against $Cp_2Fe^{0/+}$ using ferrocene as internal standard. Mass spectra were recorded on a Waters Autospec Premier (with Agilent 7890B GC, HR-EI-MS).

Optical Measurements

UV-vis spectra were recorded using a Perkin Elmer Lambda 465 photometer with deuterium and tungsten lamps. Fluorescence spectra were recorded on a LS 50 B luminescence spectrometer from PerkinElmer. UV/Vis and fluorescence spectra were measured in quartz glass cuvettes with 1 cm path length. A quartz cuvette with a Rydberg-Schlenk attachment was used to maintain an inert set-up. Photoluminescence quantum yields (Φ) were absolutely determined using a calibrated integrating sphere setup from Hamamatsu (Quantaurus-QY C11347-11). All measurements were performed at 25 °C using 10 mm x 10 mm long neck quartz cuvettes filled with degassed solutions of the radicals in the respective solvents. Solvents were degassed prior to use (freeze-pump-thaw). The fluorescence lifetimes (τ) of the

deoxygenated radical solutions were recorded on a FLS 920 (Edinburgh Instruments) equipped with Czerny-Turner double monochromators, an Edinburgh Instruments EPLED-375 or 405 (picosecond pulsed light emitting diode) for excitation at 375 or 405 nm, and a Hamamatsu R3809U-50 multi-channel plate (MCP) detector (detection range 250–850 nm, instrument response time <70 ps). Before each measurement, the instrument response function (IRF) was measured and considered for the evaluation of the resulting fluorescence decay curves. The lifetime measurements were analyzed with Edinburgh Instruments FAST Software and fitted using a reconvolution fit. The lifetimes could be evaluated bi- or triexponentially with a reduced X^2 between 1.17 and 1.20.

Loading of polytyrene nanoparticles with 3Cl12F·

Preparation of radical-stained PS-NPs

Carboxylated polystyrene nanoparticles (PSNPs) 200 nm were bought from Kisker Biotech (Lot Nr.:GK0006530421) and were used without any further modification. In order, to incorporate the 3Cl12F-Trityl radical, a modified protocol previously optimized for the loading of PSNPs with hydrophobic dyes was used.[18]

600 uL of the PSNP stock solution (50 mg/L) was filled into a 2 mL Eppendorf vial and diluted with 1 mL of MilliQ-water. After centrifugation at 21,130 crf for 20 min the supernatant was removed and 600 uL of MilliQ-water was added. Using this prepared stock solution, 60 µL of PSNP stock solution were added to 540 µL of MilliQ-water in individual Eppendorf tubes, resulting in 3 mg of PSNPs in each sample. After short agitation, 200 µL (THF:DMF (1:3 v/v)) of dye solution containing the corresponding amount of 3CL12F-radical (0.1 µmol;1 µmol; 4 µmol; 8 µmol; 10 µmol; 12 µmol; 14 µmol; 20 µmol) were added an again agitated on a vortex shaker. The sample was shaken for one hour on an orbital shaker (60 uu). Thereby, after intervals of 15 min, the sample was treated in an ultrasonic bath for 1-2 mins and then returned to the orbital shaker. Subsequently, 500 µL of MilliQ-water were added in each sample to stop the particle swelling process. Each sample was centrifuged at 21,130 crf for 20 min, 1 mL supernatant was removed, and 1 mL of absolute ethanol was added before the sample was sonicated for 1-2 min. This process was repeated once with ethanol and twice with water to produce a well dispersed dispersion of radical-loaded PSNPs and assure the removal of excess radicals.

For the optical measurements, 50 µL of the particle dispersion was diluted in 2950 µL MilliQ-water. All measurements were performed on a FS5 Edinburgh Instrument, utilizing an excitation wavelength of 350 nm.

Photostability Measurements

All measurements were performed on a FS5 Edinburgh Instrument. The solvent was placed in a 1-cm-optical-path-length quartz cell, purged with argon for 30 min, sealed with parafilm, and the radical was added in a concentration of about 10 µM. The sample was excited at 350 nm (excitation slit width of

5.0 nm) and the fluorescence intensity of the emission spectrum peaking at 550 nm was monitored as a function of illumination time.

Crystallographic Information

Single crystals were mounted on a 0.15 mm Mitegen micromount using perfluoroether oil. Crystal data were collected on a Bruker D8 Venture diffractometer with a Photon II area detector or Photon III area detector with CuK_α or MoK_α radiation. The structures were solved with the SHELXT[2] structure solution program using intrinsic phasing and refined with the SHELXL[3] refinement package using least squares minimizations by using OLEX2[4]

Computational Details

The *Turbomole*[5] program was used to perform structural optimizations at the unrestricted Kohn-Sham DFT level, using the B3LYP[26-29] hybrid functional with the basis sets def2-SVP or def2-TZVPP[29] and applying Grimme`s dispersion correction with Becke-Johnson damping (D3(BJ))[6,7] and the resolution of identity (RI)[8] approximation to reduce computational costs. Minima at the potential energy surfaces were validated by normal mode analysis. For the TD-DFT calculations geometry optimizations were performed on the UB3YLP/6-311+G(2d,p) level of theory using the ORCA 6.0.0 program package,[9-16] the optimized structure were proven to be real minima by the absence of imaginary frequencies in the frequency calculation. Time-dependent density functional theory (TD-DFT) at the UB3LYP/6-311+G(2d,p) level of theory was used to investigate the electronic transitions of absorption and emission spectra. The vibrational fine structure of the emission spectrum was calculated using the ORCA Excited State Dynamic (ESD) module.[16,17]

2. Syntheses, structure-analytical, and spectroscopical data

2.1. $C(C_6F_5)_3OH$

Fine Mg powder (2.43 g, 100 mmol, 3 eq.) was suspended in diethyl ether (150 mL) and cooled to 0 °C. Bromopentafluorobenzene (24.7 g, 100 mmol, 3 eq.) was added dropwise and the mixture was brought to room temperature. After 4 hours, methyl chloroformate (3.15 g, 33 mmol, 1 eq.) was added dropwise and the reaction mixture was stirred for 36 hours at room temperature, followed by 4 hours stirring under reflux conditions. The obtained dark brown reaction mixture was afterwards treated with diluted HCl solution (10 %, 20 mL) and then extracted with diethyl ether (3 x 30 mL). The collected organic phases were washed with dist. water (3 x 30 mL) and Brine solution (3 x 30 mL). After drying with $MgSO_4$, all volatiles were removed under reduced pressure and the resulting dark brown oil was then refined via fractionated sublimation. The first fraction (50 °C, $1*10^{-3}$ mbar) is the side product decafluorobenzophenone. The product is obtained at 100 °C and $1*10^{-3}$ mbar as light beige crystals (10.28 g, 54%).

^1H NMR (400 MHz, $CDCl_3$, 22 °C): δ = 4.29 (s, 1H) ppm.

^{19}F NMR (377 MHz, $CDCl_3$, 22 °C): δ = −140.0 (m, 6 *ortho*–F), −151.0 (m, 3 *para*–F), −160.2 (m, 6 *meta*–F) ppm.

2.2 $C(C_6F_5)_3Cl$

Tris(pentafluorophenyl)methanol $C(C_6F_5)_3OH$ (3.53 g, 7 mmol) was dissolved in thionyl chloride (25.00 g) resulting in a yellow solution. Pyridine (0.49 g, 7 mmol, 1 eq.) and dimethylformamide (0.52 g, 7 mmol, 1 eq.) were added and the mixture was stirred under reflux at 80 °C for 48 hours. Afterwards, the mixture was brought to room temperature and decanted on ice water. Small portions of saturated $NaHCO_3$ solution were added until the formation of gas stopped. It was then extracted with dichloromethane (3 x 30 mL) and the collected organic phases were subsequently washed with saturated $NaHCO_3$ (3 x 30 mL) and Brine solution (3 x 30 mL). Afterwards, it was dried with $MgSO_4$ and concentrated under reduced pressure. The crude product was washed with *n*-pentane and finally recrystallized in *n*-heptane. The product was obtained as a beige powder (1.43 g, 33%).

^{19}F NMR (377 MHz, $CDCl_3$, 22 °C): δ = −135.5 (m, 6 *ortho*–F), −150.0 (m, 3 *para*–F), −160.3 (m, 6 *meta*–F) ppm.

2.3. 3Cl12F·

$GaCl_3$ (0.019 g, 0.110 mmol, 1 eq.) was dissolved in SO_2ClF (2 mL) and cooled to −80 °C. Tris(pentafluorophenyl)-methyl chloride (0.06 g, 0.109 mmol) was added resulting in an immediate colour change to berry-red. The reaction mixture was stirred for 10 minutes. Afterwards, TMSCl (0.05 ml, 0.383 mmol, 3.5 eq.) was added and the reaction mixture was warmed to −30 °C and stirred for 30 minutes. During that time the colour changes from berry-red to violet. An excess of zinc powder

(0.5 g) was added and the reaction mixture was brought to room temperature immediately and stirred for 5 hours. During that time the solution turns bright orange-red and becomes fluorescent. Afterwards all volatiles were removed *in vacuo* and the residue was washed with *n*-pentane (2x). Finally, the red solution was concentrated under reduced pressure to give **3Cl12F·** as an orange-red powder (0.058 g, 0.104 mmol, 96%). Single crystals suitable for X-ray diffraction were grown from concentrated solutions in *n*-heptane.

Alternative method:

Instead of SO_2ClF one can also use *ortho*-difluorobenzene as a solvent. $GaCl_3$ (0.019 g, 0.110 mmol, 1 eq.) was dissolved in *ortho*-difluorobenzene (2 mL) and cooled to −40 °C. Tris(pentafluorophenyl)-methyl chloride (0.06 g, 0.109 mmol) was added resulting in an immediate colour change to berry-red. The reaction mixture was stirred for 5 minutes. Afterwards, TMSCl (0.05 ml, 0.383 mmol, 3.5 eq.) was added and the reaction mixture was warmed to –30 °C and stirred for 30 minutes. During that time the colour changes from berry-red to violet. Zinc powder (0.5 g) was added and the reaction mixture was brought to room temperature immediately and stirred for 12 hours. During that time the solution turns bright orange-red and becomes fluorescent. Afterwards the suspension is filtered and the residue washed several times with *n*-pentane (2x). The obtained orange-red solution is concentrated *in vacuo* and the crude product is purified by column chromatography (silica gel, cyclohexane) to give **3Cl12F·** as an orange-red powder (0.053 g, 0.095 mmol, 87%).

UV-Vis ($CHCl_3$, 22 °C): λ_{abs} = 347 nm. ε_{347nm} = 3.2 · 10^4 M^{-1} cm^{-1}.

Fluorescence ($CHCl_3$, 22 °C): λ_{em} = 549 nm.

EPR (toluene, 22 °C): g_{iso} = 2.004.

IR (ATR, 22 °C): $\tilde{\upsilon}$ = 1466.4 (s), 1395.1 (m), 1370.5 (m), 962.6 (vs), 899.4 (m), 781.1 (m), 675.1 (m), 668.9 (m) cm^{-1}.

Mass (EI, positive mode) m/z: [M]+ Calcd for $C_{19}F_{12}Cl_3$ 560.8873; Found 560.8692.

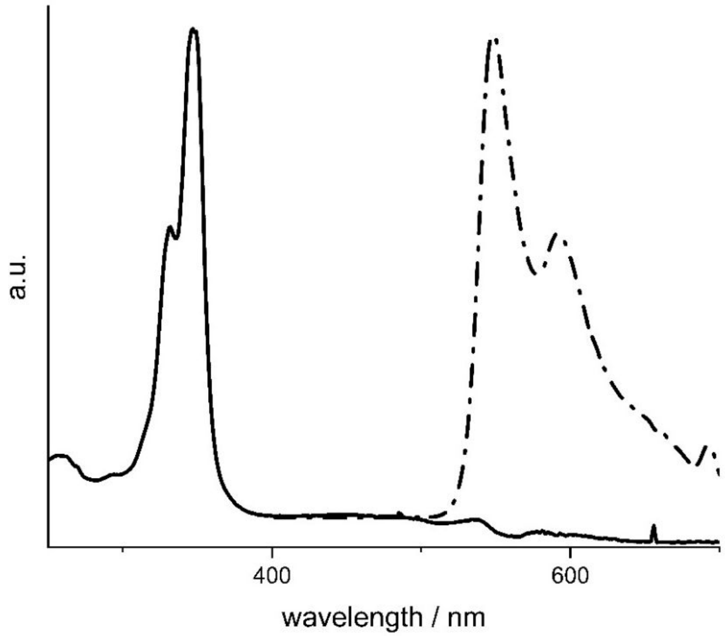

Figure S 1: Normalized UV-Vis absorption and normalized fluorescence emission spectra of 3Cl12F· in deaerated $CHCl_3$ at 22 °C. Excitation was at 350 nm.

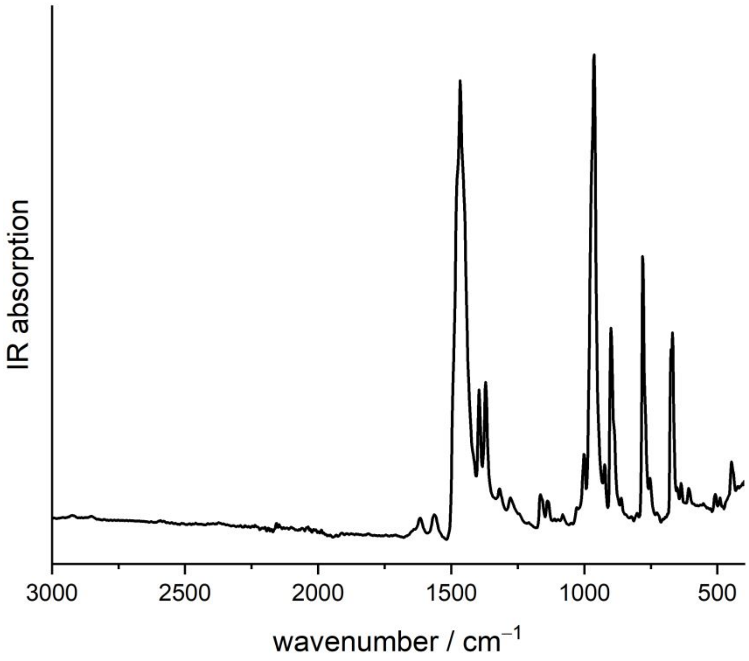

Figure S 2: IR (ATR) of 3Cl12F· at 22 °C.

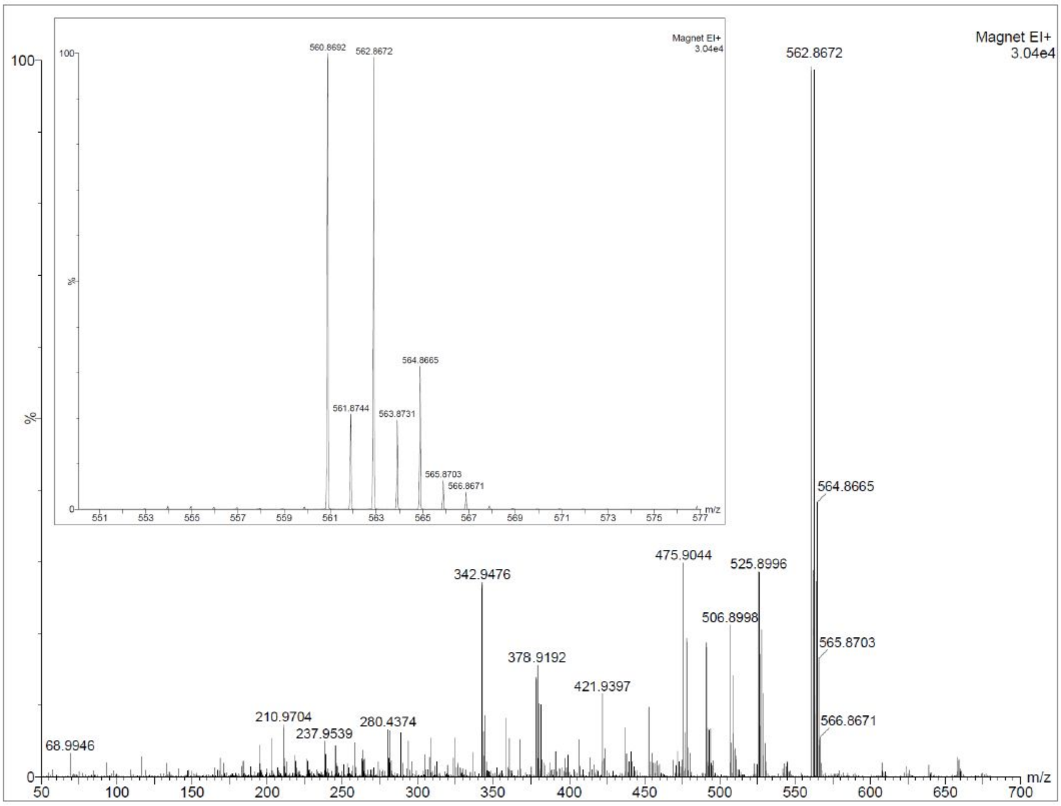

Figure S 3: Mass spectrum (EI, positive mode) of **3Cl12F·**.

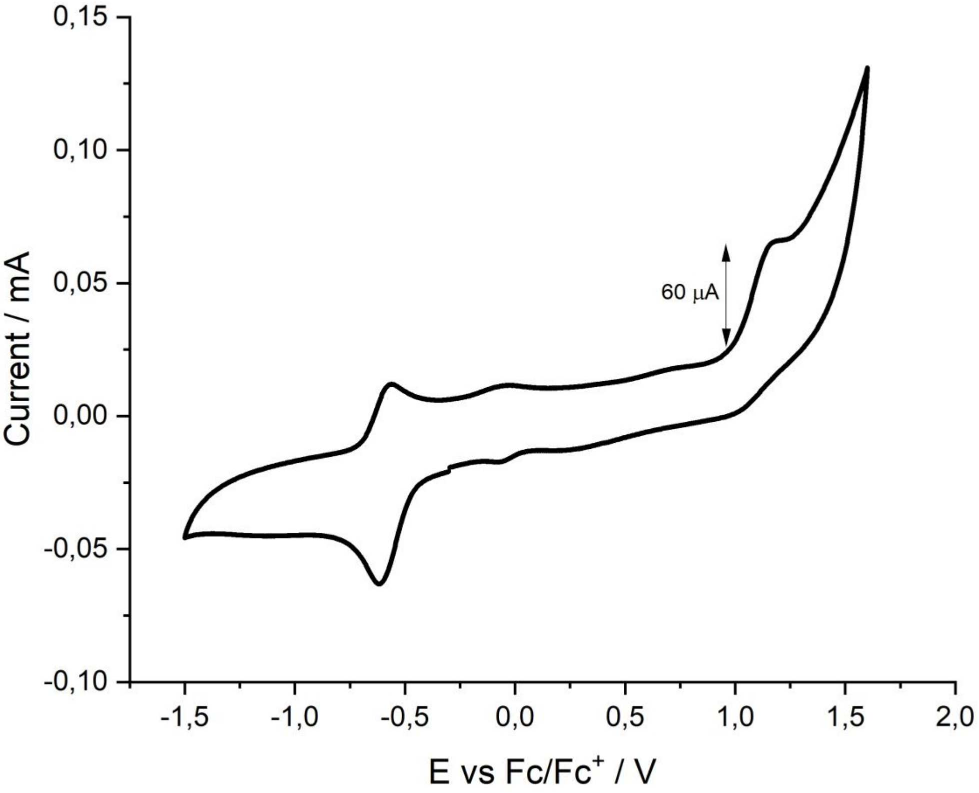

Figure S 4: Cyclic voltammogram of **3Cl12F·** in methylene chloride at 298 K (0.1 M NBu_4PF_6, 100 mVs^{-1}).

2.4. Scale-up

The herein described reactions are readily scalable, and various batch sizes between 0.1 mmol and 1.0 mmol have been tested. In the following, the synthesis of **3Cl12F·** on a larger scale is described.

Synthesis of **3Cl12F·** on a 1.0 mmol scale.

$GaCl_3$ (0.1761 g, 1.0 mmol, 1 eq.) was dissolved in *ortho*-difluorobenzene (17 mL) and cooled to –40 °C. Tris(pentafluorophenyl)-methyl chloride (0.5486 g, 1.0 mmol, mmol) was added resulting in an immediate colour change to berry-red. The reaction mixture was stirred for 5 minutes. Afterwards, TMSCl (0.44 ml, 3.5 mmol, 3.5 eq.) was added and the reaction mixture was warmed to –30 °C and stirred for 30 minutes. During that time the color changes from berry-red to violet. Zinc powder (2.0 g) was added and the reaction mixture was brought to room temperature immediately and stirred for 12 hours. During that time the solution turns bright orange-red and becomes fluorescent. Afterwards the suspension is filtered and the residue washed (2x) with *n*-pentane. The obtained orange-red solution is concentrated *in vacuo* and the crude product is purified by column chromatography (silica gel, cyclohexane) to give **3Cl12F·** as an orange-red powder (0.51 g, 0.91 mmol, 91%).

2.5. 3Br12F·

$GaCl_3$ (0.019 g, 0.110 mmol, 1 eq.) was dissolved in SO_2ClF (2 mL) and cooled to –80 °C. Tris(pentafluorophenyl)-methyl chloride (0.06 g, 0.109 mmol) was added resulting in an immediate colour change to berry-red. The reaction mixture was stirred for 10 minutes. Afterwards, TMSBr (0.05 ml, 0.383 mmol, 3.5 eq.) was added and the reaction mixture was warmed to –30 °C and stirred for 30 minutes. During that time the colour changes from berry-red to blue. Zinc powder (0.5 g) was added and the reaction mixture was brought to room temperature immediately and stirred for 5 hours. During that time the solution turns dark red and becomes fluorescent. Afterwards all volatiles were removed *in vacuo* and the residue was washed with *n*-pentane (2x). Finally, the red solution was concentrated under reduced pressure to give **3Br12F·** as a dark red powder (0.07 g, 0.102 mmol, 94%). Single crystals suitable for X-ray diffraction were grown from concentrated solutions in *n*-heptane.

Alternative method:

Instead of SO_2ClF one can also use *ortho*-difluorobenzene as a solvent. $GaCl_3$ (0.019 g, 0.110 mmol, 1 eq.) was dissolved in *ortho*-difluorobenzene (2 mL) and cooled to –40 °C. Tris(pentafluorophenyl)-methyl chloride (0.06 g, 0.109 mmol) was added resulting in an immediate colour change to berry-red. The reaction mixture was stirred for 5 minutes. Afterwards, TMSBr (0.05 ml, 0.383 mmol, 3.5 eq.) was added and the reaction mixture was warmed to –30 °C and stirred for 30 minutes. During that time the colour changes from berry-red to blue. Zinc powder (0.5 g) was added and the reaction mixture was brought to room temperature immediately and stirred for 12 hours. During that time the solution turns dark red and becomes fluorescent. Afterwards the suspension is filtered and the residue washed several times with *n*-pentane (2x). The obtained red solution is concentrated *in vacuo* and the crude product is

purified by column chromatography (silica gel, cyclohexane) to give **3Br12F·** as a dark red powder (0.065 g, 0.095 mmol, 78%).

UV-Vis ($CHCl_3$, 22 °C): λ_{abs} = 350 nm. $\varepsilon_{350nm} = 3.5 \cdot 10^4$ M^{-1} cm^{-1}.

Fluorescence ($CHCl_3$, 22 °C): λ_{em} = 547 nm.

EPR (toluene, 22 °C): g_{iso} = 2.006.

IR (ATR, 22 °C): $\tilde{\upsilon}$ = 1466.4 (s), 1390.9 (m), 1370.5 (m), 999.3 (w), 960.6 (vs), 923.9 (m), 899.4 (m), 887.2 (w), 872.9 (w), 854.5 (w), 777.1 (m), 752.6 (w), 666.9 (m), 632.2 (w), 603.7 (w), 489.4 (w), 479.3 (w), 469.1 (w), 446.6 (w), 420.1 (w), 403.8 (w) cm^{-1}.

Mass (EI, positive mode) m/z: [M]+ Calcd for $C_{19}F_{12}Br_3$ 692.7358; Found 692.7235.

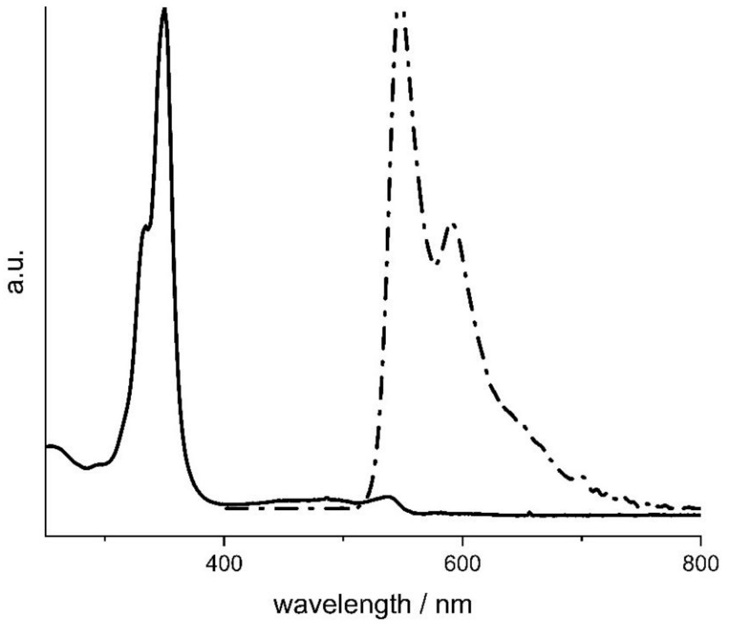

Figure S 5: Normalized UV-Vis absorption and normalized fluorescence emission spectra of 3Br12F· in deaerated $CHCl_3$ at 22 °C. Excitation was at 350 nm.

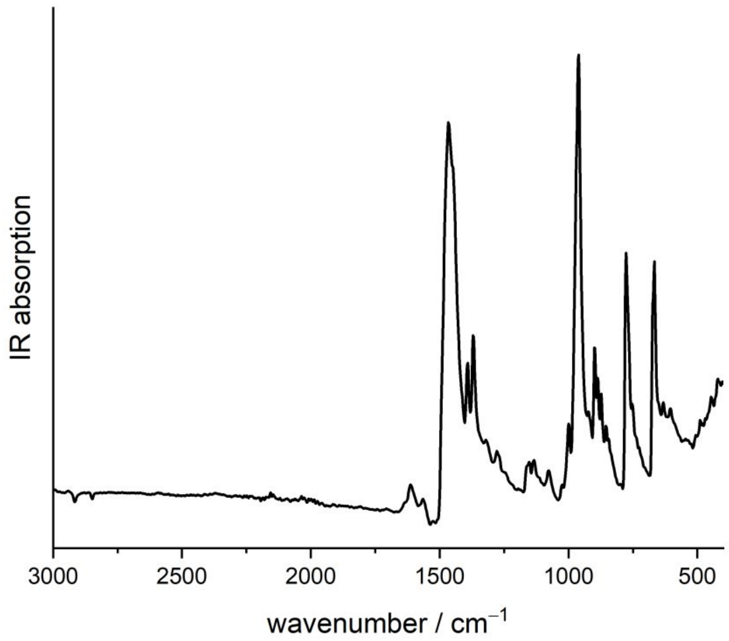

Figure S 6: IR (ATR) of 3Br12F· at 22 °C.

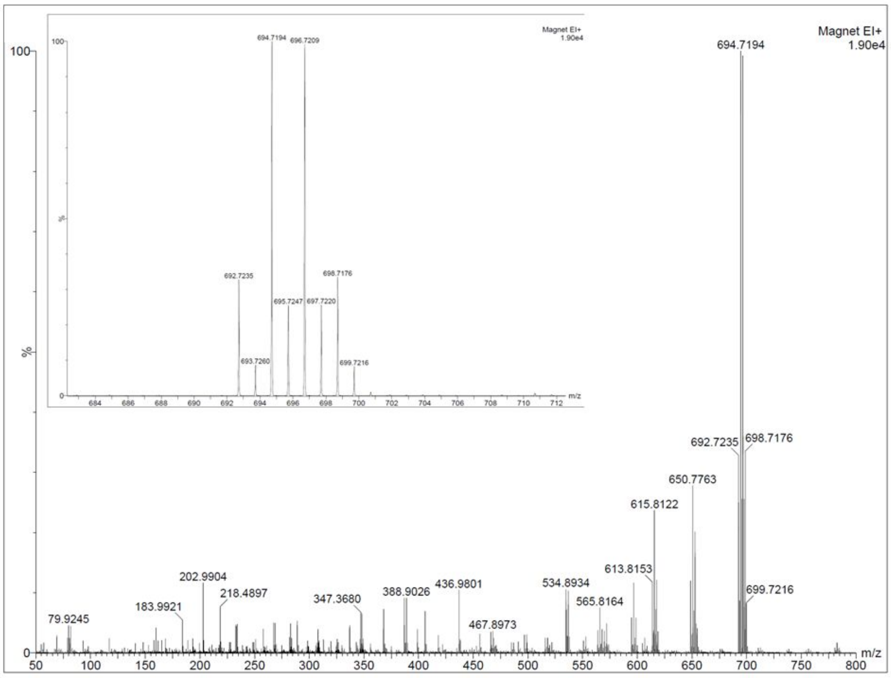

Figure S 7: Mass spectrum (EI, positive mode) of **3Br12F·**.

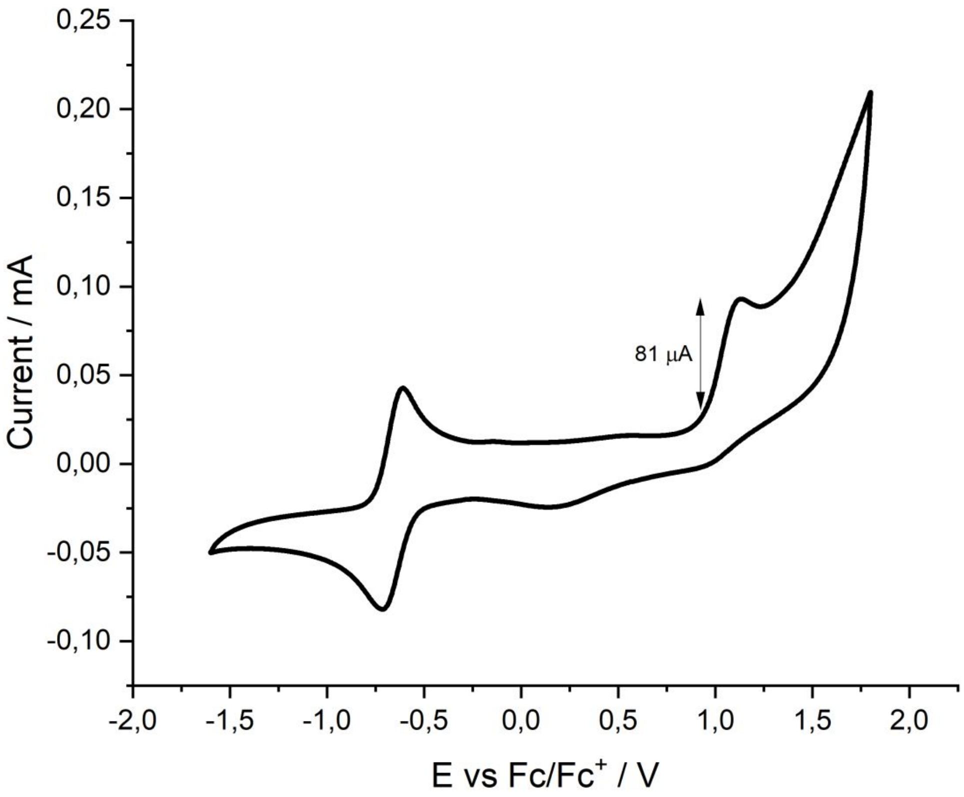

Figure S 8: Cyclic voltammogram of **3Br12F·** in methylene chloride at 298 K (0.1 M NBu_4PF_6, 100 mVs^{-1}).

2.6. [9Cl6F][Ga_2Cl_7]

$GaCl_3$ (0.038 g, 0.219 mmol, 2 eq.) was dissolved in SO_2ClF (2 mL) and cooled to –80 °C. Tris(pentafluorophenyl)-methyl chloride (0.06 g, 0.109 mmol) was added resulting in an immediate colour change to berry-red. The reaction mixture was stirred for 10 minutes. Afterwards, TMSCl (0.4 ml, 0.383 mmol, 30 eq.) was added and the reaction mixture was brought to room-temperature immediately and stirred for 5-7 days. During that time the colour changes from berry-red to blue.

^{19}F NMR (377 MHz, SO_2ClF, ext. acetone-d_6, –80 °C): δ = –140.4 (s, 6 *meta*–F) ppm.

^{13}C {^{19}F} NMR (101 MHz, SO_2ClF, ext. acetone-d_6, –80 °C): δ = 145.7 (*ortho*–C), 144.4 (*meta*–C), 145.6 (*para*–C), 114.6 (*ipso*–C) ppm.

UV-Vis (SO_2ClF/*o*-DFB, –40 °C): λ_{abs} = 638 nm.

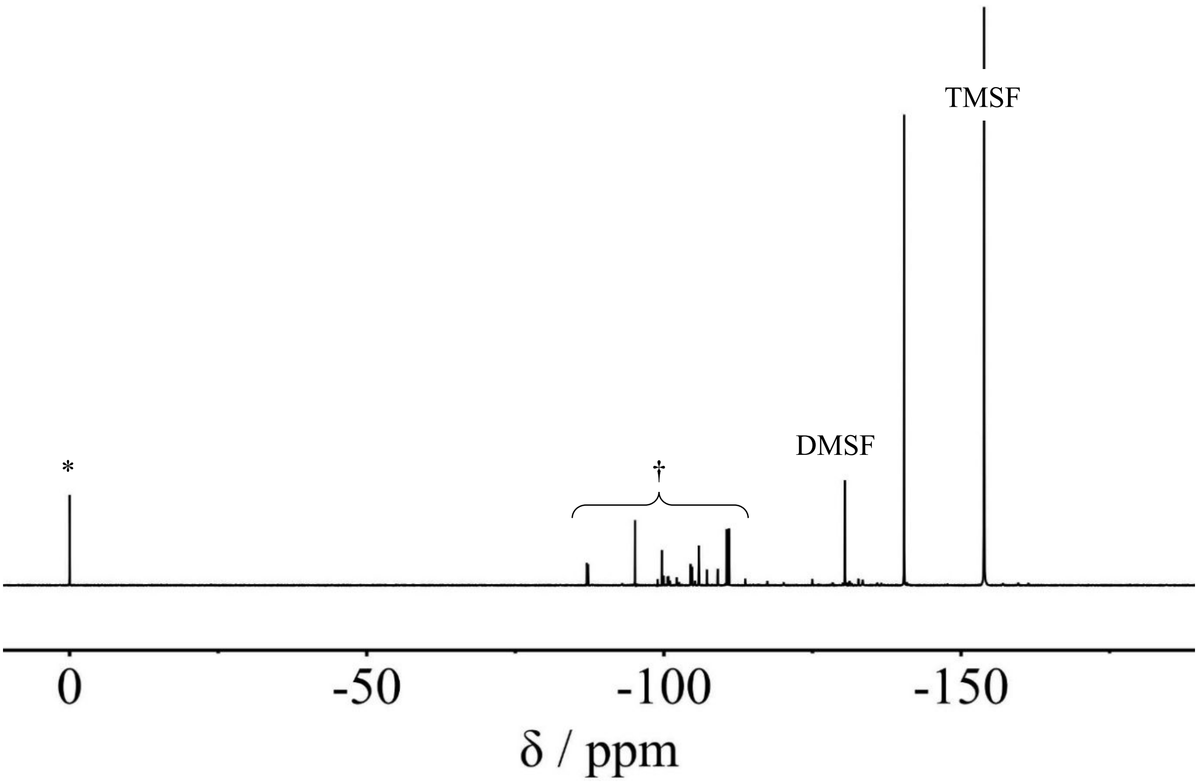

Figure S 9: ^{19}F NMR (377 MHz, SO_2ClF, ext. acetone–d_6, −80 °C) of the reaction mixture in the synthesis of [9Cl6F][Ga_2Cl_7]. The asterisk marks the signal of the acetone-d_6 capillary. The cross marks unidentified side-products. DMSF = dimethylsilylfluoride

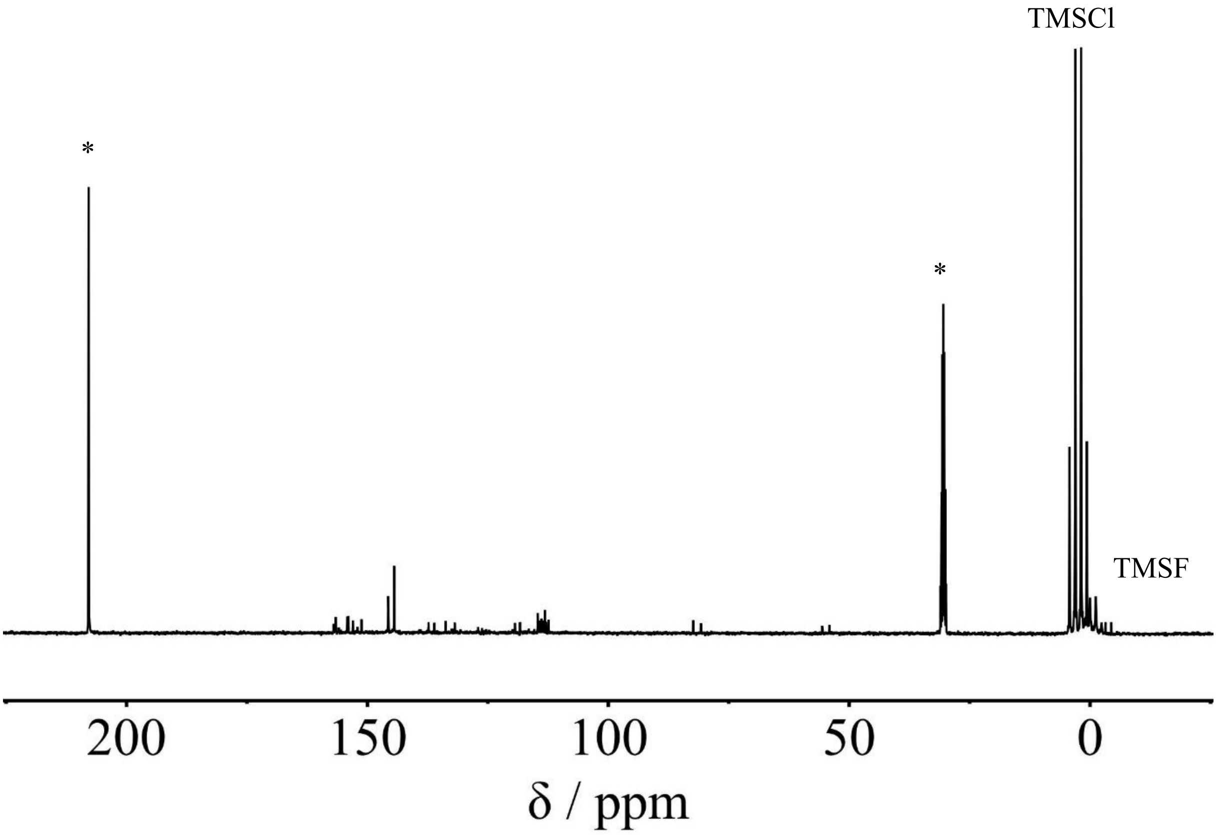

Figure S 10: ^{13}C {^{19}F} NMR (101 MHz, SO_2ClF, ext. acetone-d_6, −80 °C) of the reaction mixture in the synthesis of [9Cl6F][Ga_2Cl_7]. The asterisks mark the signal of the acetone-d_6 capillary.

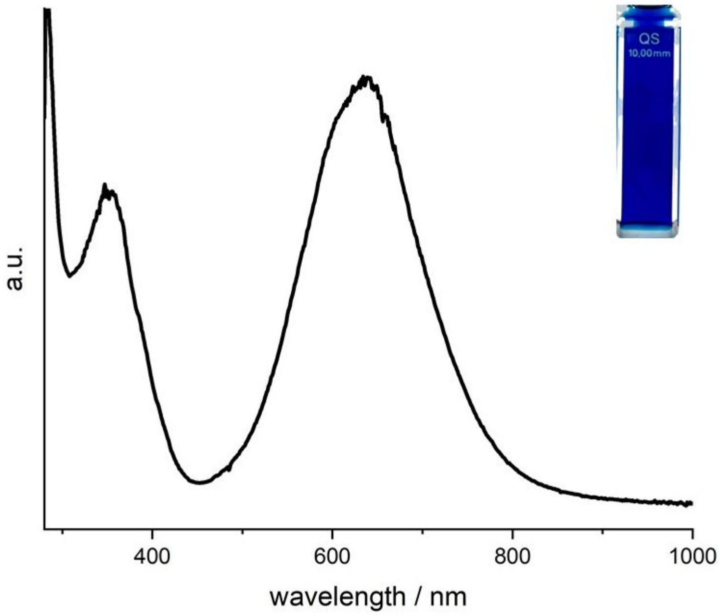

Figure S 11: Normalized UV-Vis spectrum of the reaction mixture in the synthesis of [9Cl6F][Ga_2Cl_7] in SO_2ClF/*o*-DFB at –40 °C.

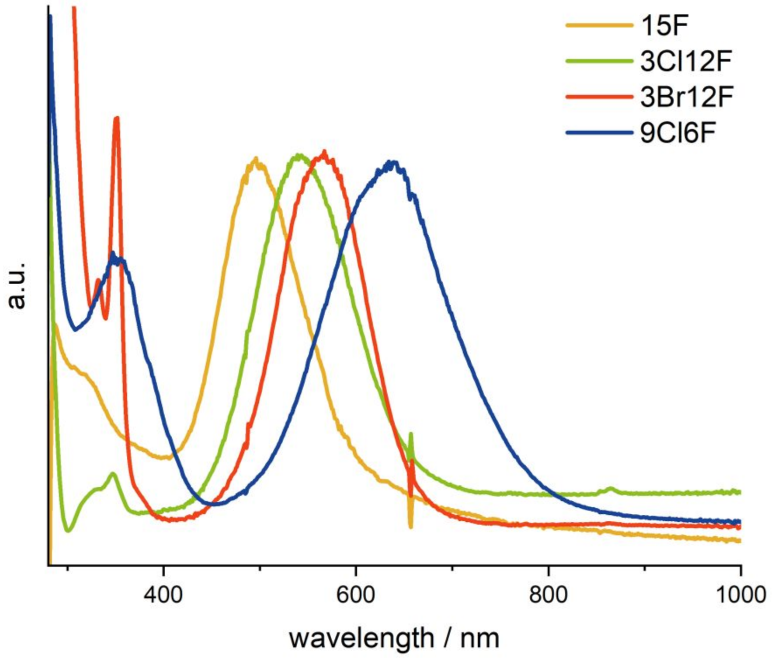

Figure S 12: Normalized UV-Vis spectra of different perhalofluoro trityl cations in SO_2ClF/*o*-DFB at −40 °C. The spectra were always normalized at the respective absorption maximum.

Table S 1: HOMO-LUMO gaps of different perhalofluoro tritylcations calculated at the B3LYP-D3(BJ)/def2-TZVPP level of theory.

	$\Delta E_{HOMO\text{-}LUMO}$ /eV
$15F^+$	2.65
p-$3Cl12F^+$	2.55
$9Cl6F^+$	2.31

2.7. 9Cl6F·

$GaCl_3$ (0.038 g, 0.219 mmol, 2 eq.) was dissolved in SO_2ClF (2 mL) and cooled to −80 °C. Tris(pentafluorophenyl)-methyl chloride (0.06 g, 0.109 mmol) was added resulting in an immediate colour change to berry-red. The reaction mixture was stirred for 10 minutes. Afterwards, TMSCl (0.4 ml, 0.383 mmol, 30 eq.) was added and the reaction mixture was brought to room-temperature immediately and stirred for 5-7 days. During that time the colour changes from berry-red to blue. Zinc powder (0.5 g) was added at −30 °C and the reaction mixture was brought back to room temperature and stirred for 12 hours. During that time the solution turns dark red and becomes fluorescent. Afterwards all volatiles were removed *in vacuo* and the residue was washed with *n*-pentane (2x). Finally, the red solution was concentrated under reduced pressure and the crude product is purified by column chromatography (silica gel, cyclohexane) to give **9Cl6F·** as a bright red powder (0.031 g, 0.047 mmol, 43%). Single crystals suitable for X-ray diffraction were grown from concentrated solutions in *n*-heptane.

UV-Vis ($CHCl_3$, 22 °C): λ_{abs} = 370 nm. ε_{370nm} = 1.9 · 10^4 M^{-1} cm^{-1}.

Fluorescence ($CHCl_3$, 22 °C): λ_{em} = 587 nm.

EPR (toluene, 22 °C): $g_{iso} = 2.004$.

IR (ATR, 22 °C): ῦ = 1484.8 (vs), 1466.4 (s), 1429.7 (m), 1401.2 (m), 1368.6 (w), 1337.9 (w), 1278.8 (w), 1070.7 (w), 1005.5 (m), 968.8 (s), 915.7 (w), 885.1 (m), 836.2 (w), 801.5 (w), 783.1 (w), 770.9 (m), 738.3 (m), 709.7 (w), 668.9 (m), 650.6 (m), 601.6 (w), 446.6 (w) cm^{-1}.

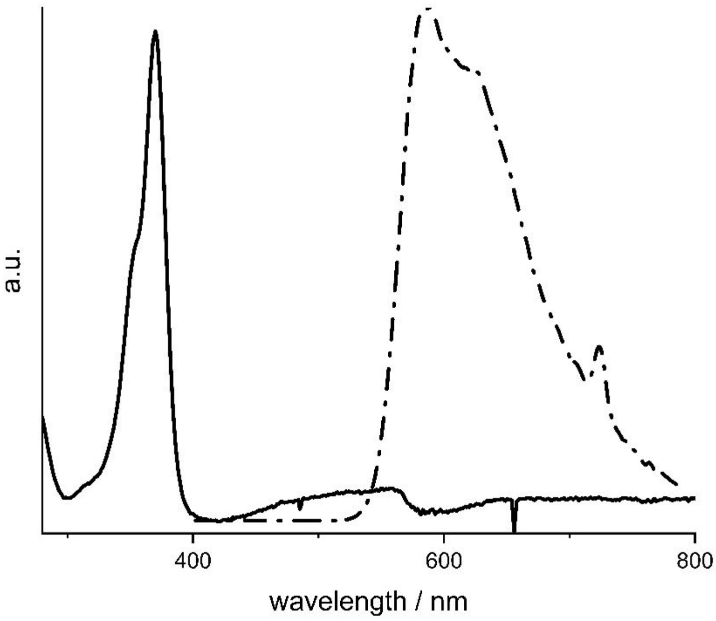

Figure S 13: Normalized UV-Vis absorption and normalized fluorescence emission spectra of 9Cl6F· in deaerated $CHCl_3$ at 22 °C. Excitation was at 370 nm.

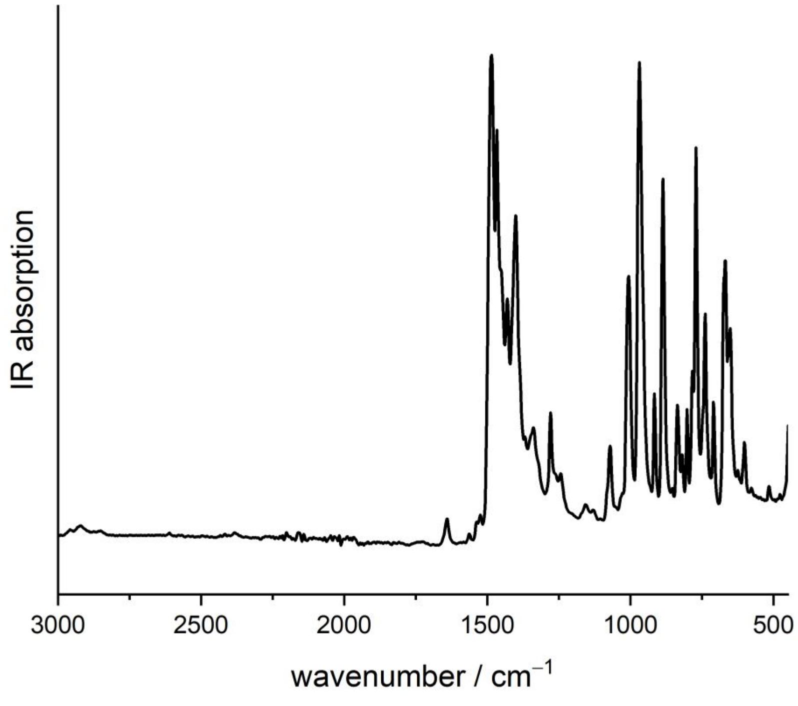

Figure S 14: IR (ATR) of 9Cl6F· at 22 °C.

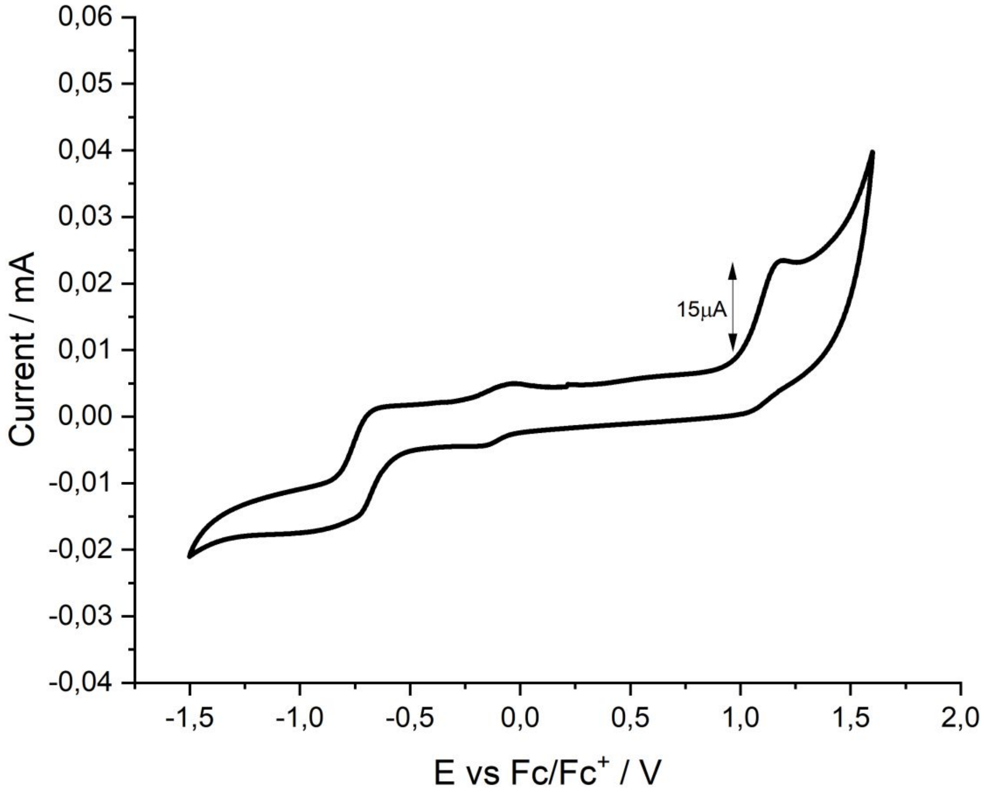

Figure S 15: Cyclic voltammogram of **9Cl6F·** in methylene chloride at 298 K (0.1 M NBu_4PF_6, 100 mVs^{-1}).

2.8. $(3Cl12FO)_2$

In a beaker C(*para*-$ClC_6F_4)_3$ was dissolved in *n*-heptane and left at ambient conditions for 7 days until the solution turns light yellow. During that time the crude product precipitates as a colourless powder. Afterwards all volatiles are removed *in vacuo*, the residue is washed with dichloromethane and concentrated under reduced pressure to give $(3Cl12FO)_2$ as a colourless powder. Single crystals suitable for X-ray diffraction were grown from concentrated solutions in *n*-heptane.

^{19}F NMR (377 MHz, CH_2Cl_2, ext. acetone-d_6, 22 °C): δ = −135.7 (d, 6 *ortho*–F), −140.5 (d, 6 *meta*–F) ppm.

^{13}C {^{19}F} NMR (101 MHz, CH_2Cl_2, ext. acetone-d_6, 22 °C): δ = 144.9 (*ortho*–C), 144.5 (*meta*–C), 114.9 (*para*–C), 114.6 (*ipso*–C), 84.0 (central C) ppm.

Raman (CH_2Cl_2, −196 °C): ṽ = 1643 (w), 1261 (m), 891 (s), 519 (w) cm^{-1}.

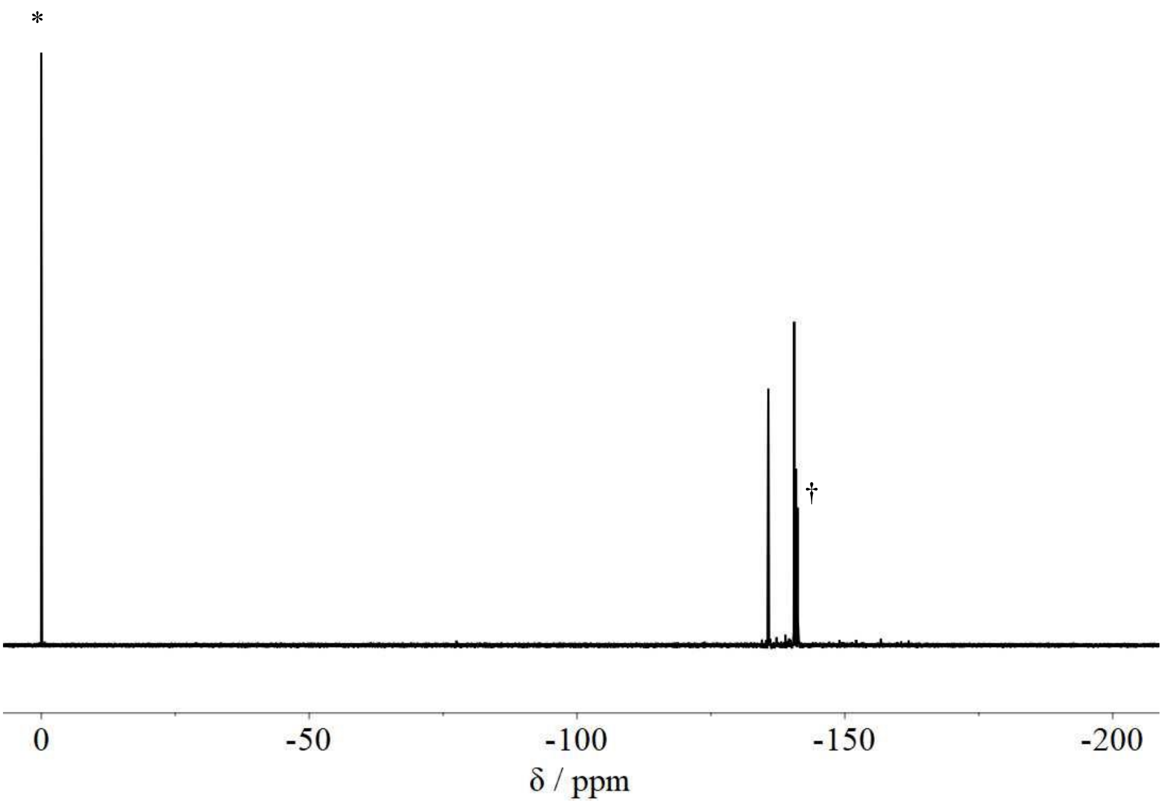

Figure S 16: ^{19}F NMR (377 MHz, CH_2Cl_2, ext. acetone–d_6, 22 °C) of $(3Cl12FO)_2$. The asterisk marks the signal of the acetone-d_6 capillary. The cross marks unidentified impurity.

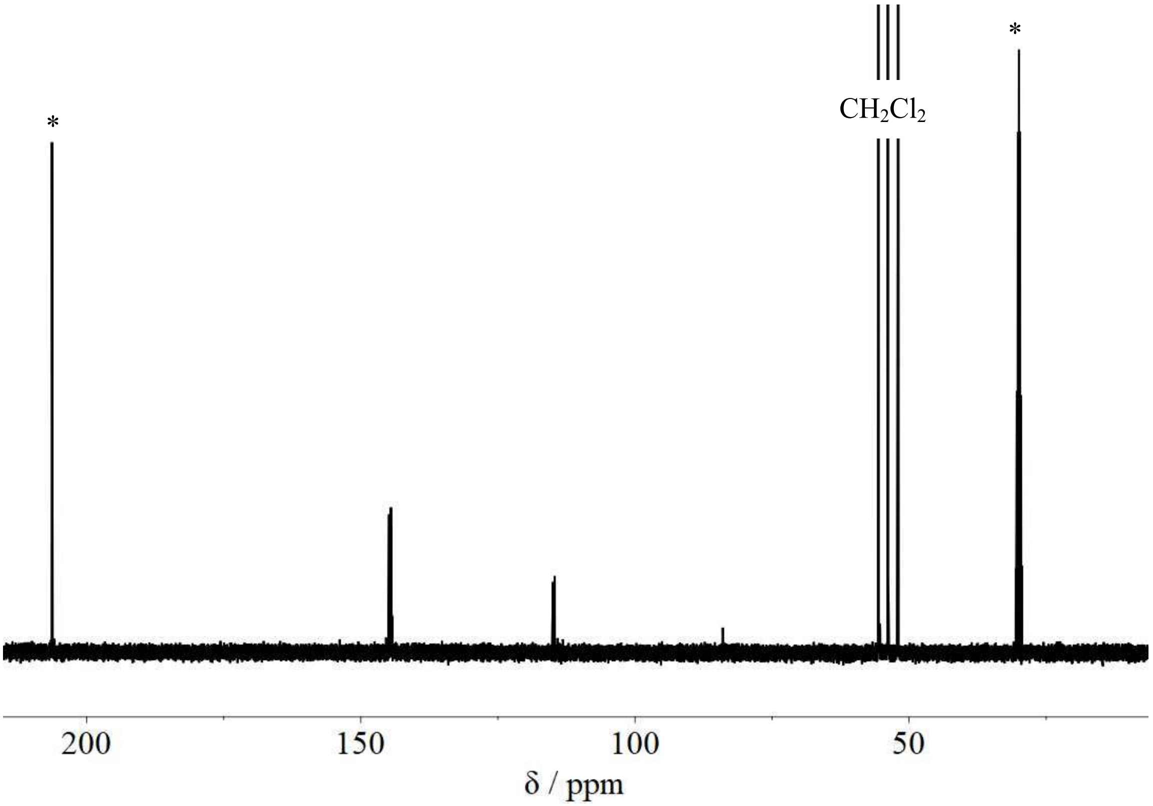

Figure S 17: ^{13}C {^{19}F} NMR (101 MHz, CH_2Cl_2, ext. acetone-d_6, 22 °C) of $(3Cl12FO)_2$. The asterisks mark the signal of the acetone-d_6 capillary.

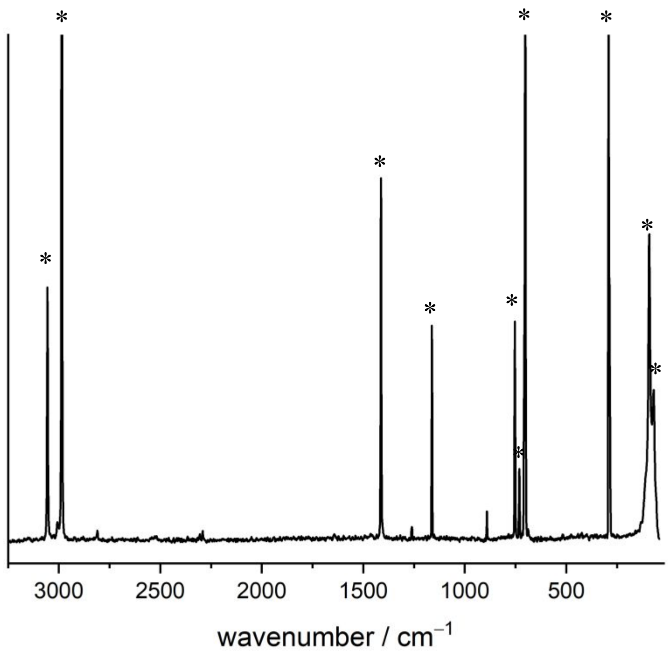

Figure S 18: Raman spectrum (CH_2Cl_2, −196 °C) of $(3Cl12FO)_2$. The asterisks mark the bands of the solvent.

2.9. $(3Br12FO)_2$

In a beaker $C(para\text{-}BrC_6F_4)_3$ was dissolved in *n*-heptane and left at ambient conditions for 7 days until the solution turns pale yellow. During that time the crude product precipitates as a colourless powder. Afterwards all volatiles are removed *in vacuo*, the residue is washed with dichloromethane and concentrated under reduced pressure to give **$(3Br12FO)_2$** as a colorless powder. Single crystals suitable for X-ray diffraction were grown from concentrated solutions in *n*-heptane.

^{19}F NMR (377 MHz, CH_2Cl_2, ext. acetone-d_6, 22 °C): δ = −133.1 (m, 6 *ortho*–F), −140.7 (m, 6 *meta*–F) ppm.

$^{13}C\{^{19}F\}$ NMR (101 MHz, CH_2Cl_2, ext. acetone-d_6, 22 °C): δ = 145.4 (*ortho*–C), 144.8 (*meta*–C), 115.6 (*ipso*–C), 102.4 (*para*–C), 84.3 (central C) ppm.

Raman (CH_2Cl_2, −196 °C): $\tilde{\nu}$ = 1640 (w), 1261 (m), 890 (s), 504 (w) cm^{-1}.

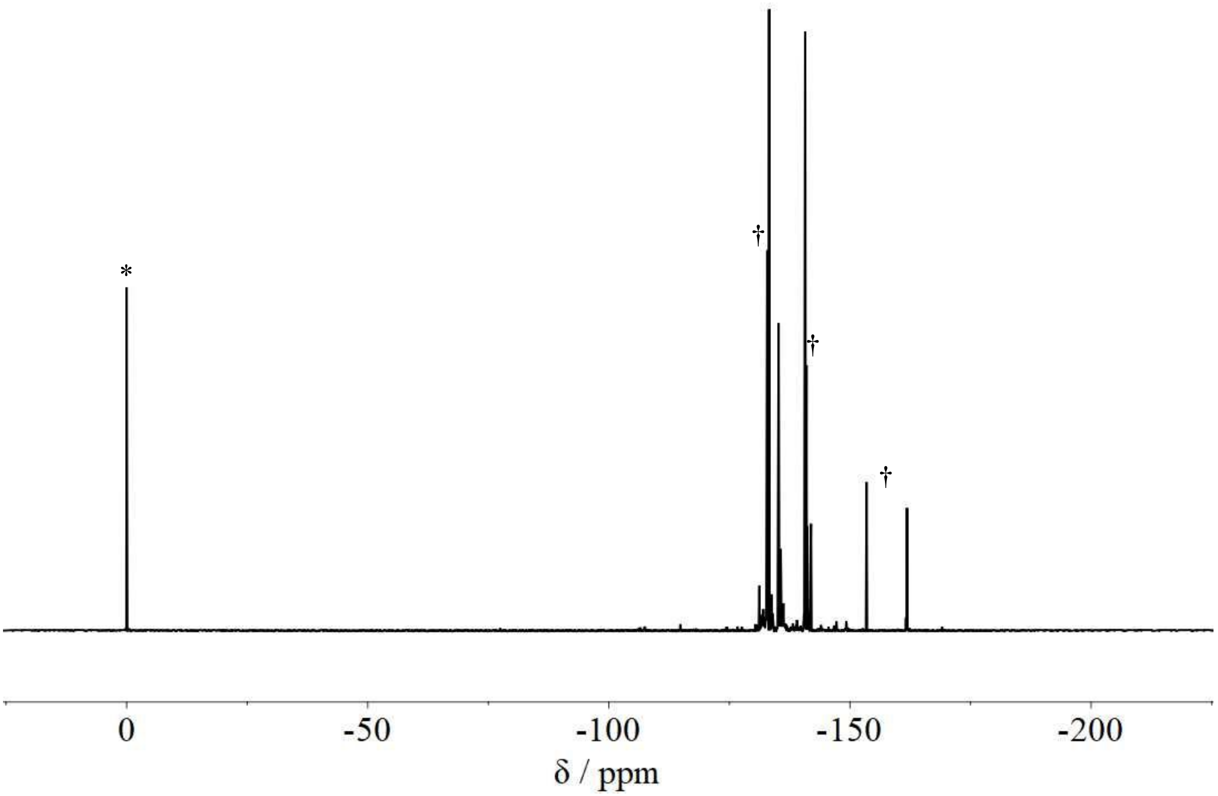

Figure S 19: ^{19}F NMR (377 MHz, CH_2Cl_2, ext. acetone–d_6, 22 °C) of $(3Br12FO)_2$. The asterisk marks the signal of the acetone-d_6 capillary. The cross marks unidentified impurity.

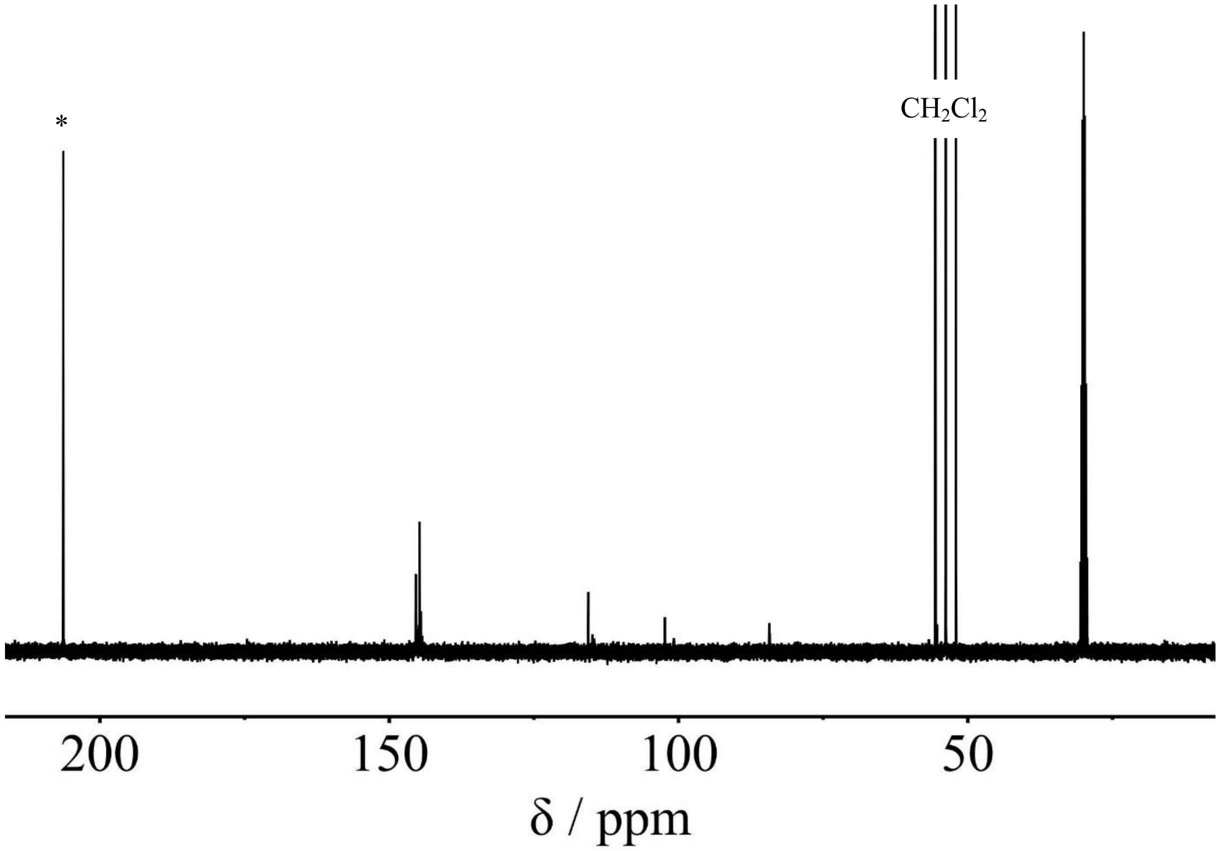

Figure S 20: ^{13}C {^{19}F} NMR (101 MHz, CH_2Cl_2, ext. acetone-d_6, 22 °C) of $(3Br12FO)_2$. The asterisks mark the signal of the acetone-d_6 capillary.

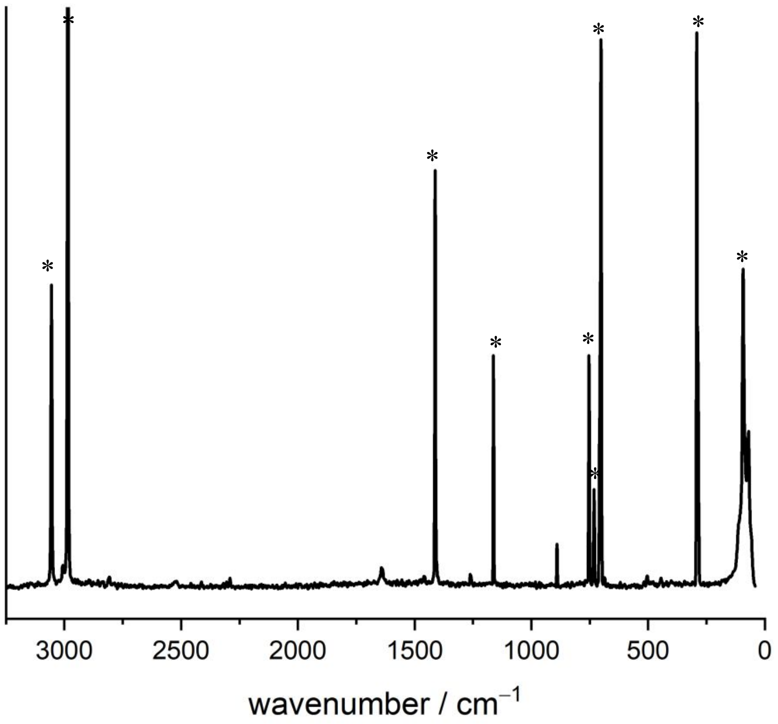

Figure S 21: Raman spectrum (CH_2Cl_2, −196 °C) of $(3Br12FO)_2$. The asterisks mark the bands of the solvent.

2.10. Stability of Perhalofluoro Trityl Radicals

In contrast to *ortho*-chlorinated radicals including **9Cl6F·**, the radicals **3X12F·** are not inert toward oxygen and degrade in solution under ambient conditions. To investigate the rate of this process in a qualitative way, a solution of **3Cl12F·** in *n*-heptane ($c = 4 \cdot 10^{-5}$ mol/l, V = 2.4 mL, $n_{3Cl12F\cdot} = 1.44 \cdot 10^{-4}$ mmol) was placed in a sealable UV-Vis cuvette ($V_{tot} = 6$ mL). *n*-Heptane was chosen due to the high solubility of oxygen in this solvent.[69] The cuvette was sealed under ambient conditions, ensuring a large excess of oxygen in the residual volume ($n_{O2} \approx 0.029$ mmol, 200 eq.). UV-Vis spectra recorded every 12 hours over seven days revealed a continuous decrease in the absorption maximum of **3Cl12F·** at 347 nm, corresponding to an 8% loss within the first 24 hours, and a 33% loss after seven days (Fig. S22).

The relatively slow reduction in absorption intensity suggests that, even though the **3X12F·** radicals are less inert than their chlorinated counterparts, they are not instantaneously quenched by O_2, but react relatively slow with oxygen. This aligns with our experience, that both *para*-halogenated trityl radicals **3X12F·** are sufficiently stable in solution to be handled under ambient conditions for at least a few hours. For example, no sign of peroxide formation is found after purification of the radicals *via* flash chromatography (if the fractions are concentrated soon after collection).

The purity of the radicals is most reliably confirmed by the absence of signals in the ^{19}F NMR spectrum or by mass spectrometry. In the latter case, a fragment corresponding to **3Cl12FO·**, indicative of the peroxide species, can be detected. Such a signal is for example observed in the mass spectrum of a powdered sample of **3Cl12F·**, that had been stored under ambient conditions for more than one year (Fig. S23).

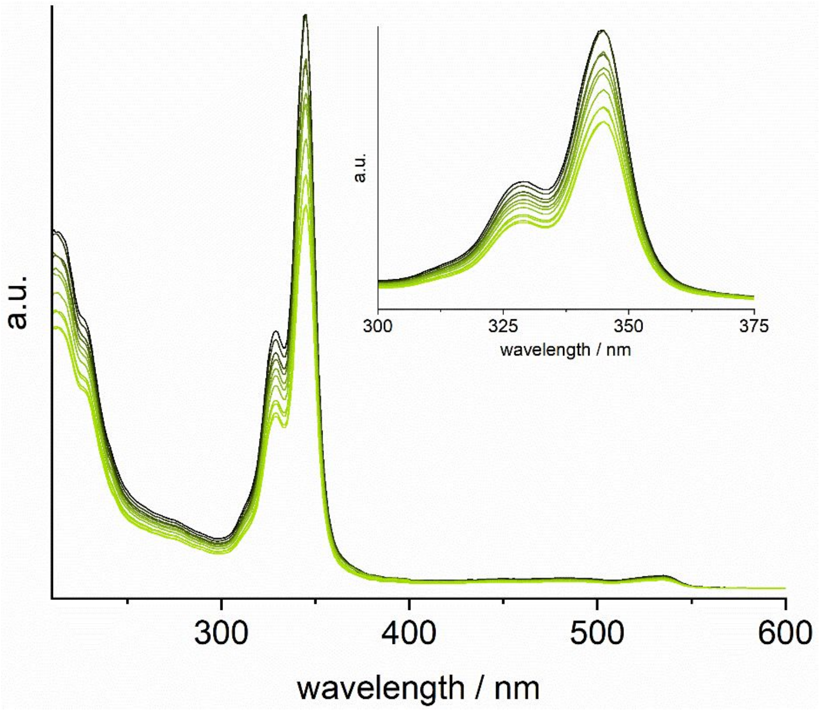

Figure S 22: UV-Vis absorption spectra of an aerated solution of 3Cl12F· in *n*-heptane at 22 °C measured at several storage intervals to monitor radical stability.

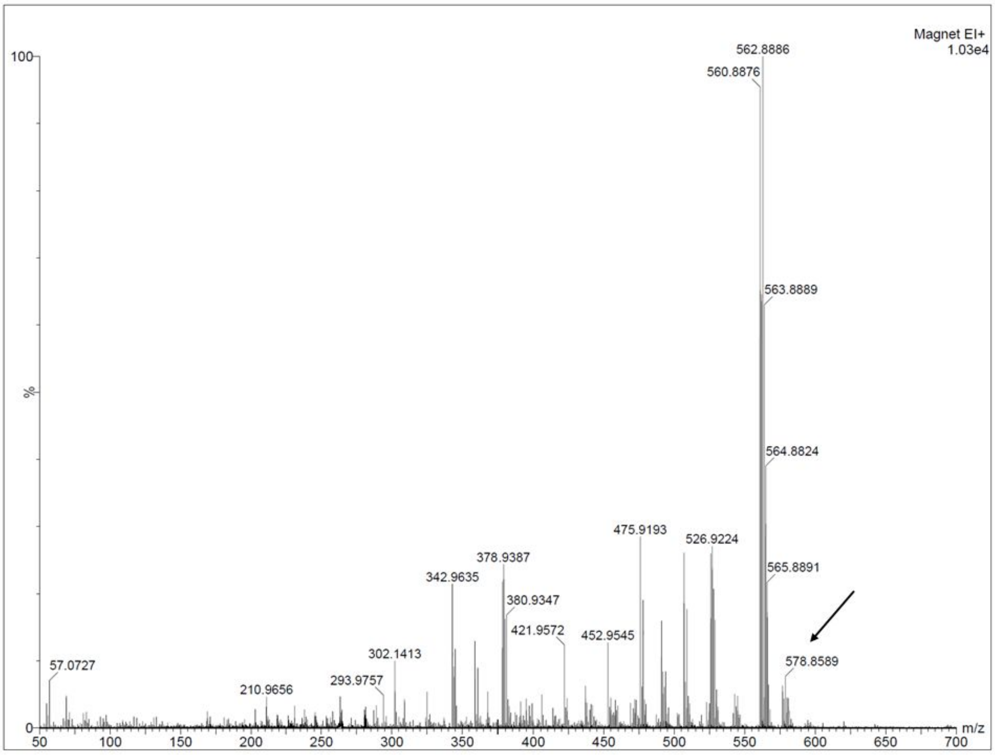

Figure S 23: Mass (EI, positive mode) of an over one year old powdered sample of 3Cl12F·. The arrow marks the signal attributed to the 3Cl12FO-fragment of the peroxide.

3. Optical Data

3.1. Luminescence properties of 3X12F· in various organic solvents

Table S 2: UV-Vis absorption and fluorescence emission spectra of **3Cl12F·** and **3Br12F·** in various solvents at 22°C. All measurements were performed with deaerated solutions (sh = shoulder).

	λ_{abs} / nm		λ_{em} / nm	
	**3Cl12F·**	**3Br12F·**	**3Cl12F·**	**3Br12F·**
Cyclohexane	331 (sh), 347	330 (sh), 351	547, 591 (sh)	550, 593 (sh)
Chloroform	330 (sh), 347	330(sh), 350	547, 590 (sh)	549, 595 (sh)
Methylene chloride	331 (sh), 347	334 (sh), 351	549, 594 (sh)	553, 595 (sh)
Toluene	332 (sh), 347	335 (sh), 351	555 (sh), 618, br	561 (sh), 615, br
Acetonitrile	329 (sh), 343	331 (sh), 348	550 (sh), 641, br	553 (sh), 652, br
Acetone	329 (sh), 344	332 (sh), 349	555 (sh), 670, br	559 (sh), 657, br
Ethanol	329 (sh), 344	333(sh), 350	553 (sh), 633, br	554 (sh), 644, br

Table S 3: Absolutely measured photoluminescence quantum yields and luminescence lifetimes for **3Cl12F·** and **3Br12F·** in various deaerated solvents at 22°C.

	Φ / %		τ / ns	
	**3Cl12F·**	**3Br12F·**	**3Cl12F·**	**3Br12F·**
Cyclohexane	9.0	8.4	20.0	27.5
Chloroform	5.6	5.9	17.0	19.8
Methylene chloride	3.2	3.7	13.1	10.8
Toluene	1.8	1.8	8.5	n.d.
Acetonitrile	1.0	1.0	4.4	n.d.
Acetone	<1.0	<1.0	n.d.	n.d.
Ethanol	<1.0	n.d.	n.d.	n.d.

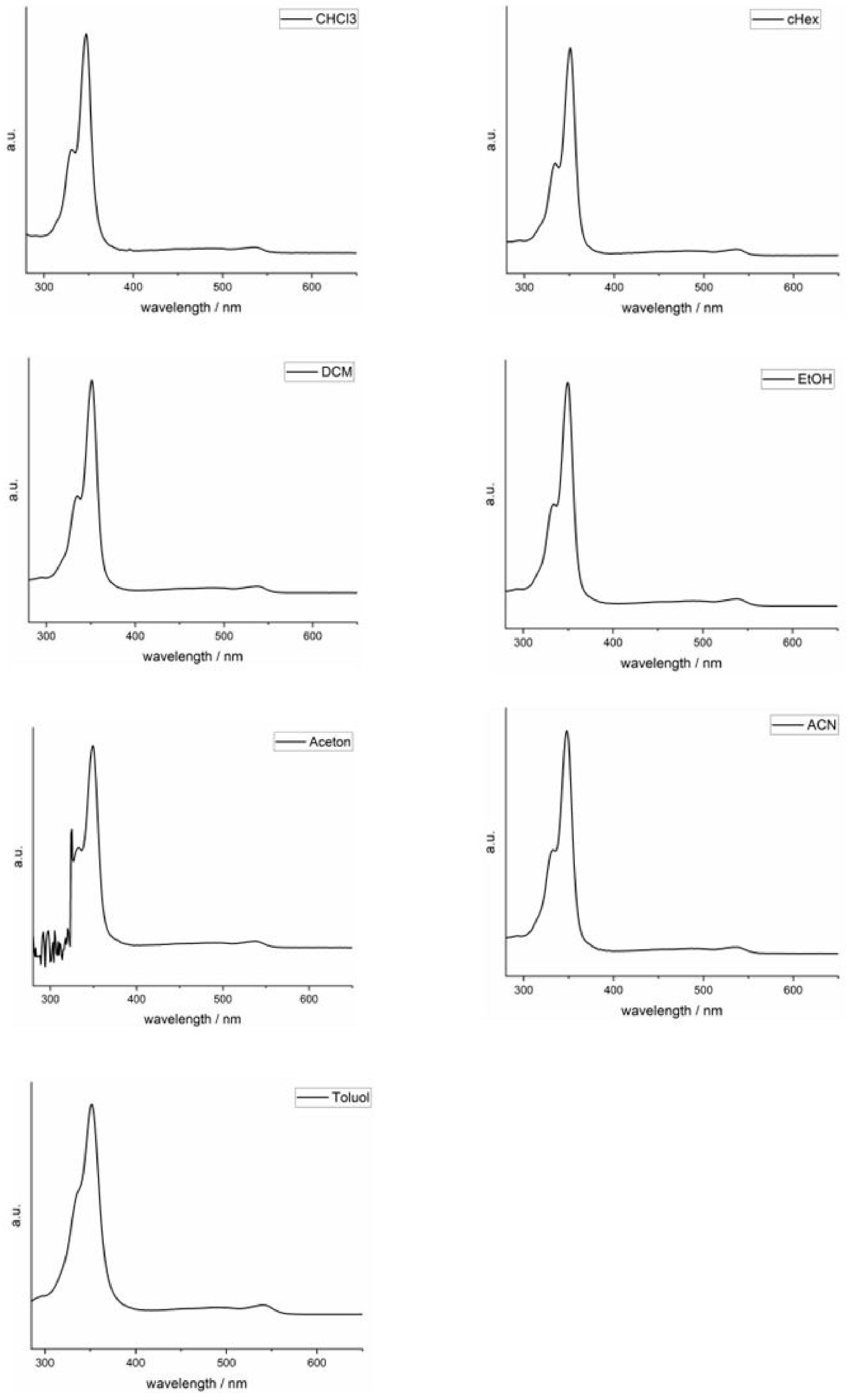

Figure S 24: Normalized UV-Vis absorption spectra of **3Br12F·** in various deaerated organic solvents at 22 °C. The spectra were normalized at the respective absorption maximum.

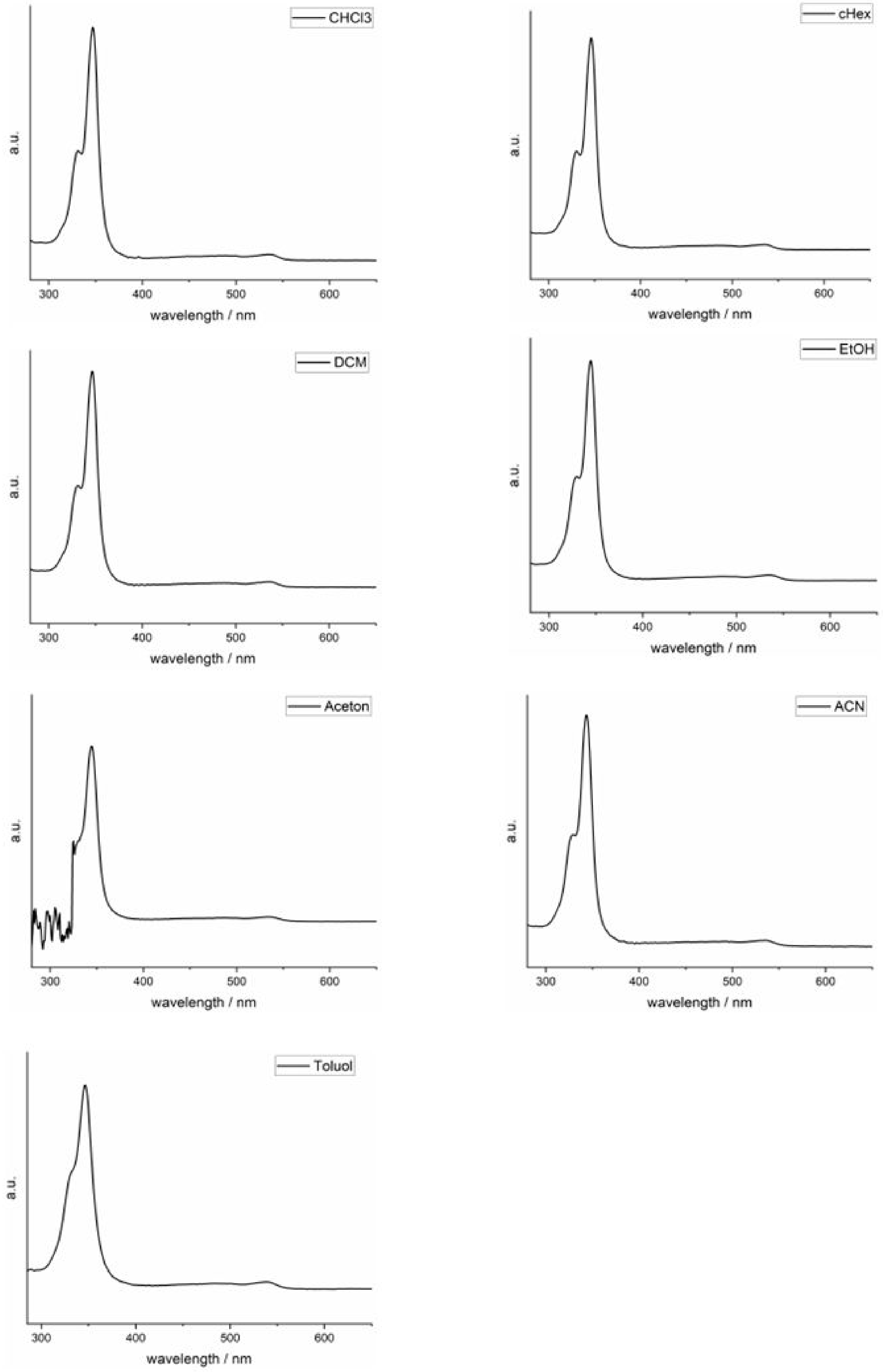

Figure S 25 : Normalized UV-Vis absorption spectra of **3Cl12F·** in various deaerated organic solvents at 22 °C. The spectra were normalized at the respective absorption maximum.

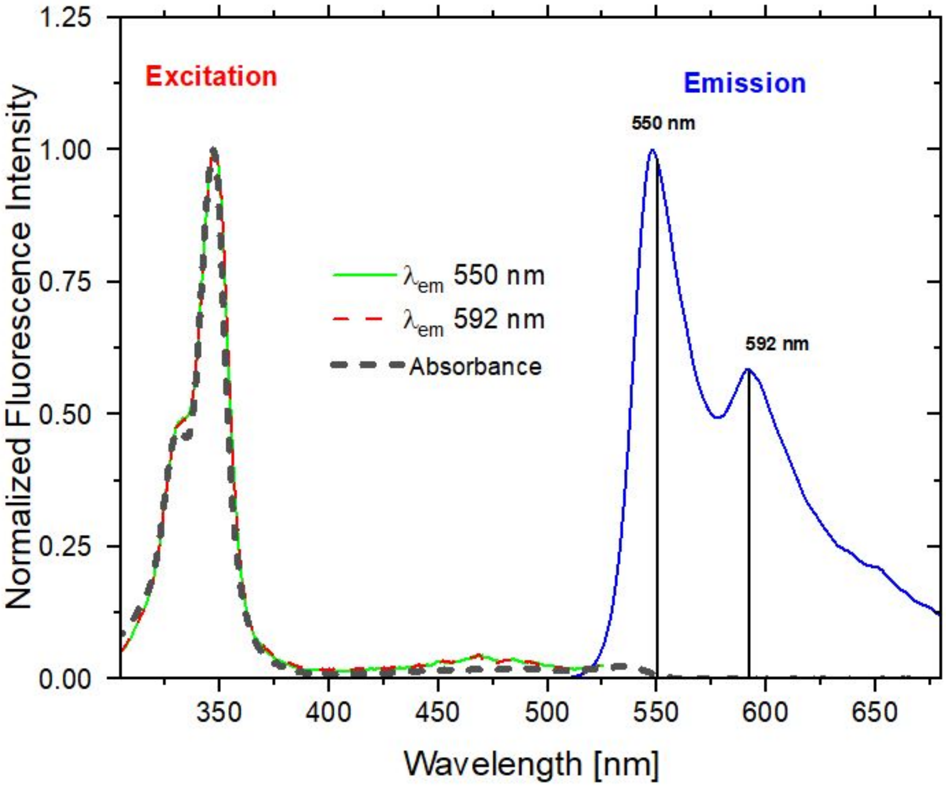

Figure S 26: Normalized fluorescence excitation spectra of Cl312F-trityl radical (concentration of 10 uM) in chloroform (Top) recorded at 550 nm and 592 nm, respectively, using an excitation slit width (ExBW) of 4 nm and an emission slit width (EmBW) of 10 nm. The respective normalized absorbance spectra (dashed) and the emission spectra (excited at 350 nm) are included as well.

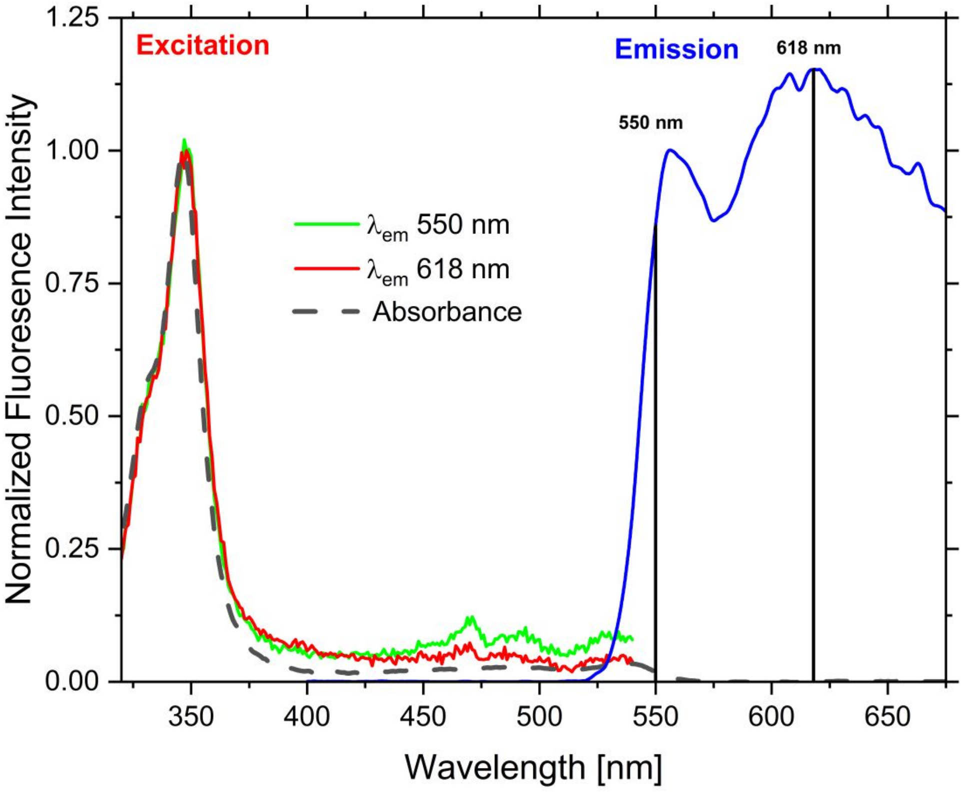

Figure S 27: Normalized fluorescence excitation spectra of Cl312F-trityl radical (concentration of 10 uM) recorded in toluene at 550 nm and 618 nm, respectively, using an excitation slit width (ExBW) of 4 nm and an emission slit width (EmBW) of 10 nm. The respective normalized absorbance spectra (dashed) and the emission spectra (excited at 350 nm) are included as well.

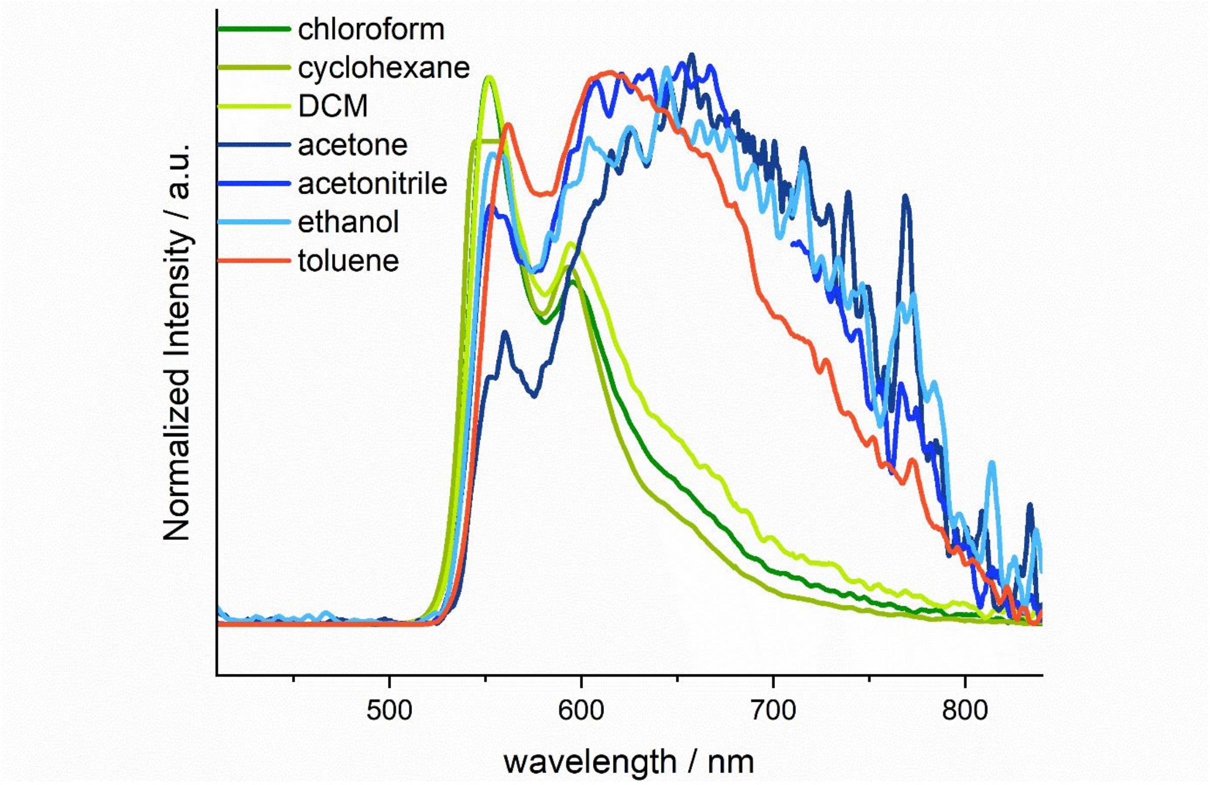

Figure S 28: Normalized Fluorescence emission spectra of **3Br12F·** in various deaerated organic solvents exited at 347 nm at 22 °C. Normalization was done at the respective emission maximum.

3.2. Photostability of 3Cl12F·

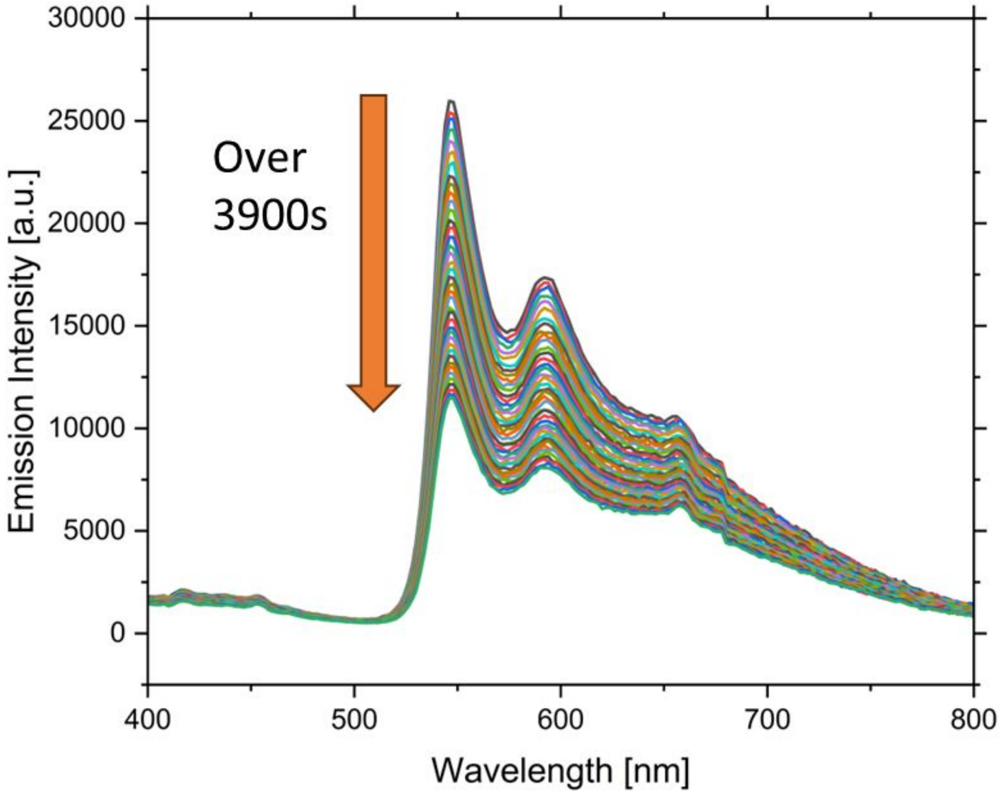

Figure S 29: Emission spectra of **3Cl12F·** in $CHCl_3$ (≈ 10 μM) at different illumination times using a fluorometer as light source. For the time-dependent emission studies, the sample was purged with argon for 30 minutes and sealed with parafilm prior to measurements, exited at 370 nm over ≈1h, each spectrum was taken after 100 s. Excitation slit width ExBw= 5 nm; emission slit width EmBw=3 nm. The temperature was kept at 20 °C (thermostate).

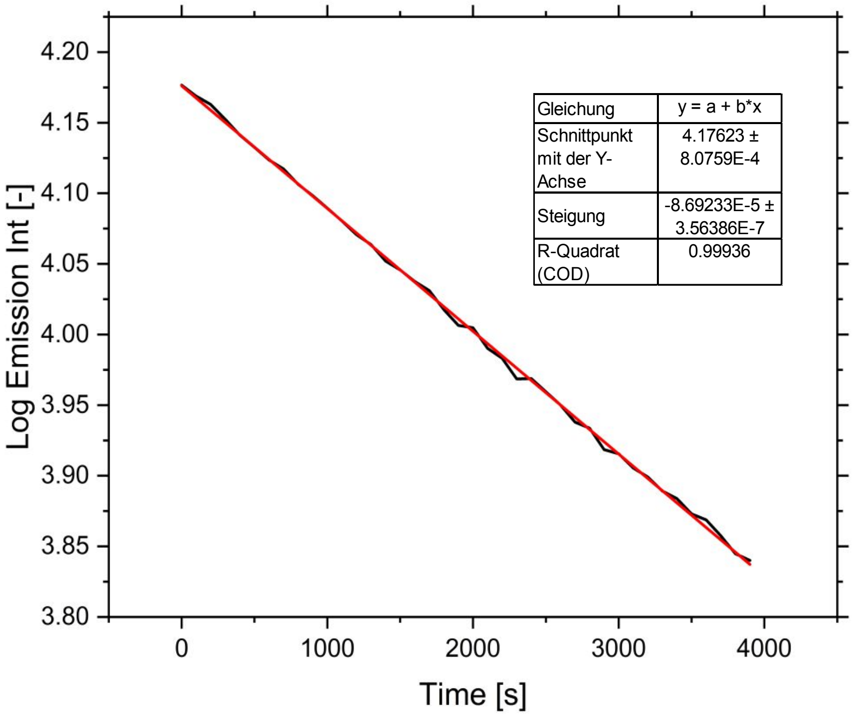

Figure S 30: Time-dependent change in emission intensity of **3Cl12F·** in $CHCl_3$ (≈ 10 µM) monitored at 570 nm following the continuous illumination at 370 nm (the corresponding emission spectra are shown in Figure S27) The intensity changes were fitted with a linear fit.

3.3. Luminescence properties of 3Cl12F· in polystyrene nanoparticles

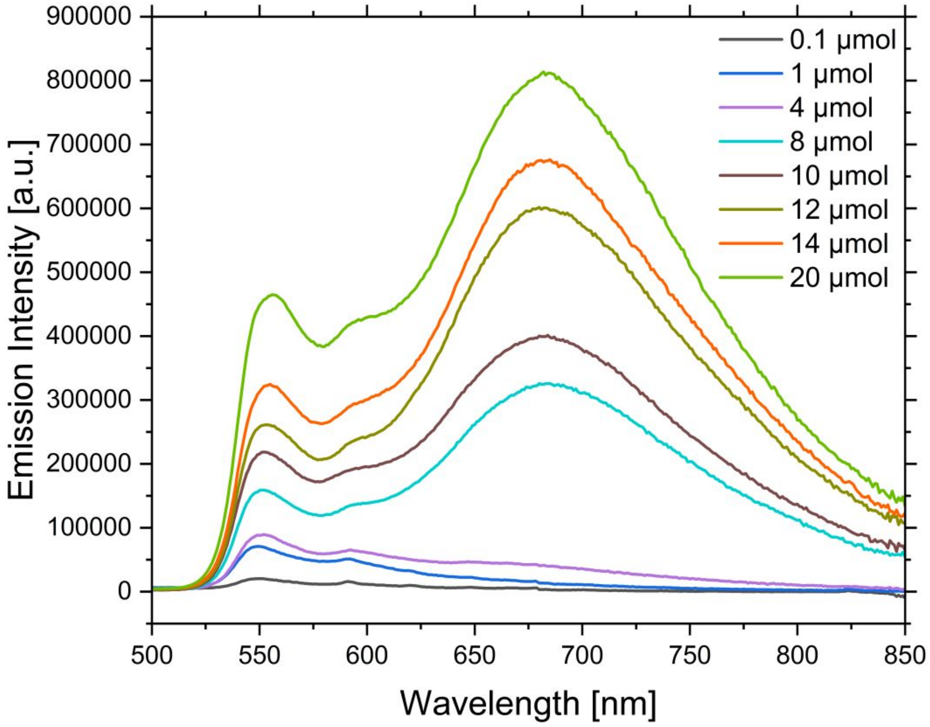

Figure S 31: Emission spectra of **3Cl12F·** in 200 nm PS-NPs obtained for loading concentrations from 0.1 µmol to 20 µmol showing the gradual emergence of a new red-shifted and broad band with a maximum at about 685 nm. (Excitation was at 350 nm).

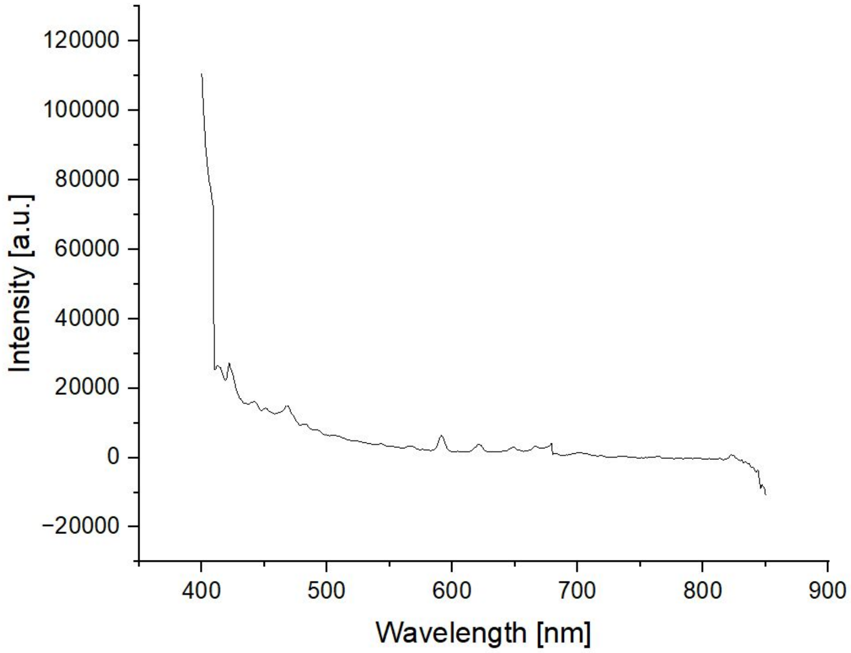

Figure S 32: Emission/Scattering spectra of neat PS-NPs used for **3Cl12F·**-loading, excitation at 350 nm.

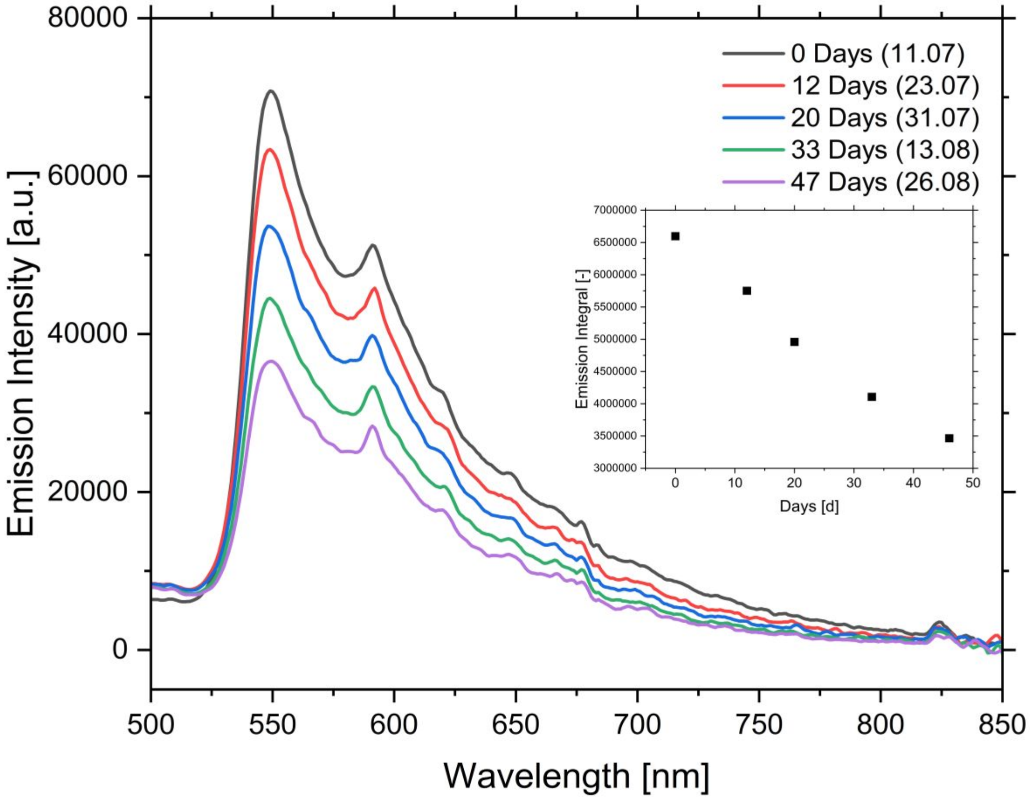

Figure S 33: Long-term stability study of **3Cl12F·** encapsulated into 200 nm PS-NPs over the course of 47 days monitored by fluorescence measurements, Loading concentration: 1 umol. (Excitation was at 350 nm) The inset shows the integral emission integrated from 520-820 nm; The samples, sealed with parafilm, were stored at 23 °C in the dark.

4. TD-DFT

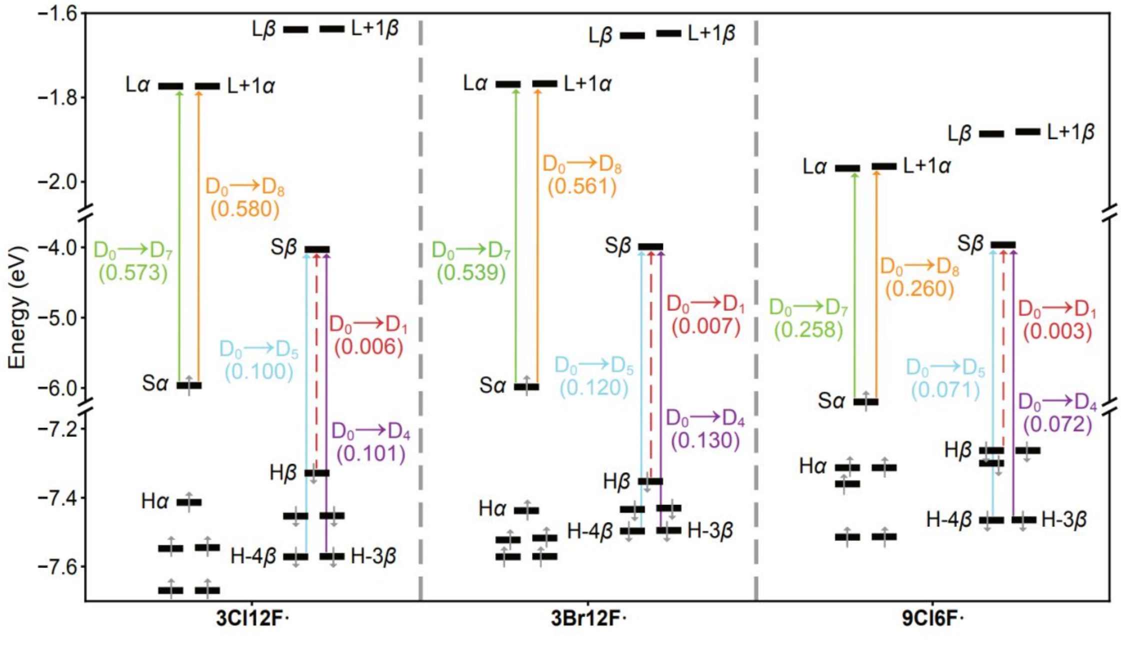

Figure S 34: Orbital diagram (HOMO (H), SOMO (S), LUMO (L)) illustrating the low oscillator strength of the *β*-excitation from $D_0 \rightarrow D_1$, and the dominant transitions $D_0 \rightarrow D_4$, $D_0 \rightarrow D_5$, $D_0 \rightarrow D_7$, and $D_0 \rightarrow D_8$ for all compounds. Oscillator strength f_{osc} given in brackets. The orbital energies were calculated at the UB3LYP/6-311+G(2d,p) level of theory.

Table S 4: Orbital contribution (HOMO (H), SOMO (S), LUMO (L)) >5%, and oscillator strength f_{osc} of electronic transitions from the ground state according to TD-DFT at the UB3LYP/6-311+G(2d,p) level of theory.

	**3Cl12F·**		**3Br12F·**		**9Cl6F·**	
Transition	Orbitals	f_{osc}	Orbitals	f_{osc}	Orbitals	f_{osc}
$D_0 \to D_1$	97% H$\beta \to$Sβ	0.006	97% H$\beta \to$Sβ	0.007	10% H-1$\beta \to$Sβ<br>83% H$\beta \to$Sβ	0.003
$D_0 \to D_2$	93% H-1$\beta \to$Sβ	0.001	94% H-1$\beta \to$Sβ	0	83% H-1$\beta \to$Sβ<br>10% H$\beta \to$Sβ	0.003
$D_0 \to D_3$	93% H-2$\beta \to$Sβ	0.006	94% H-2$\beta \to$Sβ	0	97% H-2$\beta \to$Sβ	0.016
$D_0 \to D_4$	9% S$\alpha \to$L+1α<br>81% H-3$\beta \to$Sβ	0.101	10% S$\alpha \to$L+1α<br>81% H-3$\beta \to$Sβ	0.130	84% H-3$\beta \to$Sβ	0.072
$D_0 \to D_5$	9% S$\alpha \to$Lα<br>81% H-4$\beta \to$Sβ	0.100	10% S$\alpha \to$Lα<br>82% H-4$\beta \to$Sβ	0.120	84% H-4$\beta \to$Sβ	0.071
$D_0 \to D_6$	8% S$\alpha \to$L+9α<br>80% H-5$\beta \to$Sβ	0.001	6% S$\alpha \to$L+9α<br>85% H-5$\beta \to$Sβ	0	5% S$\alpha \to$L+8α<br>87% H-5$\beta \to$Sβ	0
$D_0 \to D_7$	66% S$\alpha \to$Lα<br>15% S$\alpha \to$L+1α<br>14% H-5$\to$Sβ	0.573	81% S$\alpha \to$Lα<br>11% H-4$\to$Sβ	0.539	84% S$\alpha \to$Lα<br>6% H-4$\beta \to$Sβ	0.258
$D_0 \to D_8$	15% S$\alpha \to$Lα<br>66% S$\alpha \to$L+1α<br>14% H-3$\beta \to$Sβ	0.580	81% S$\alpha \to$L+1α<br>11% H-3$\beta \to$Sβ	0.561	84% S$\alpha \to$L+1α<br>6% H-3$\beta \to$Sβ	0.260

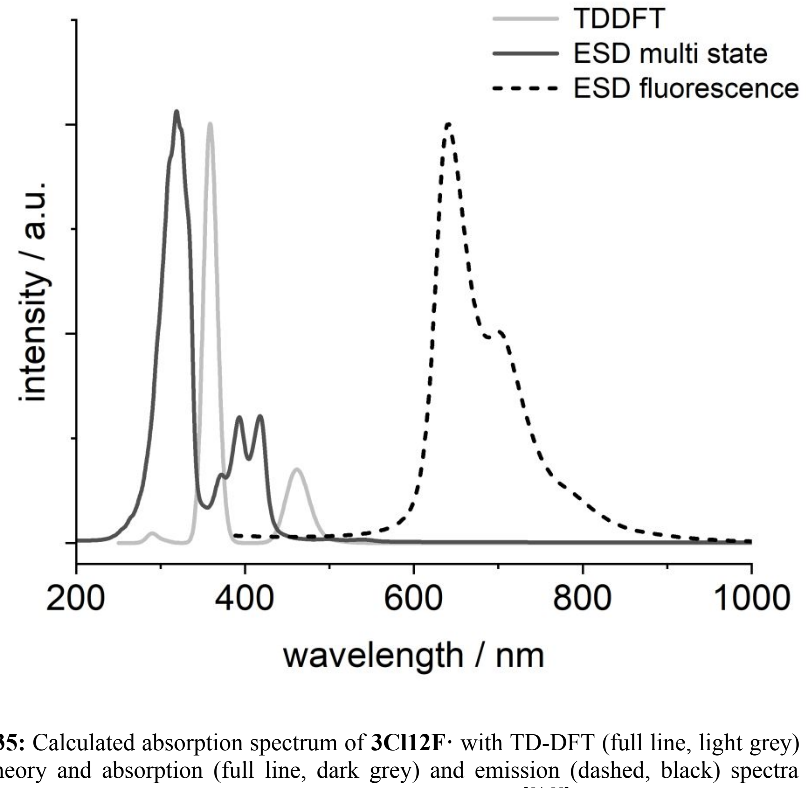

Figure S 35: Calculated absorption spectrum of **3Cl12F·** with TD-DFT (full line, light grey) at the UB3LYP/6-311+G(2d,p) level of theory and absorption (full line, dark grey) and emission (dashed, black) spectra with the ORCA Excited State Dynamics (ESD) module, accounting for vibrational broadening.[54,55]

5. Crystal Data

Summary of crystal data and refinement results

	**3Cl12F·**

CCDC number	2440952
Empirical formula	$C_{19}Cl_3F_{12}$
Formula weight	562.54
Temperature [K]	100.00
Crystal system	trigonal
Space group (number)	$R\overline{3}{:}H$ (148)
a [Å]	41.4188(16)
b [Å]	41.4188(16)
c [Å]	5.6635(3)
α [°]	90
β [°]	90
γ [°]	120
Volume [Å^3]	8414.2(8)
Z	18
ρ_{calc} [gcm^{-3}]	1.998
μ [mm^{-1}]	0.614
F(000)	4914
Crystal size [mm^3]	0.026×0.06×0.162
Crystal colour	clear light orange
Crystal shape	needle
Radiation	MoK_{α} (λ=0.71073 Å)
2θ range [°]	3.93 to 50.79 (0.83 Å)
Index ranges	−49 ≤ h ≤ 49<br>−49 ≤ k ≤ 49<br>−6 ≤ l ≤ 6
Reflections collected	50648
Independent reflections	3432<br>R_{int} = 0.1411<br>R_{sigma} = 0.0394
Completeness to θ = 25.242°	99.9 %
Data / Restraints / Parameters	3432 / 0 / 308
Absorption correction T_{min}/T_{max} (method)	0.6530 / 0.7452 (multi-scan)
Goodness-of-fit on F^2	1.143
Final *R* indexes [I≥2σ(I)]	R_1 = 0.0431<br>wR_2 = 0.0986
Final *R* indexes [all data]	R_1 = 0.0604<br>wR_2 = 0.1131
Largest peak/hole [eÅ^{-3}]	0.37/−0.42
Extinction coefficient	0.00037(5)

	**3Br12F·**
CCDC number	2440950
Empirical formula	$C_{19}Br_3F_{12}$
Formula weight	695.92
Temperature [K]	100.00
Crystal system	monoclinic

Space group (number)	$C2/c$ (15)
a [Å]	9.8138(6)
b [Å]	11.7107(7)
c [Å]	17.5868(11)
α [°]	90
β [°]	103.447(2)
γ [°]	90
Volume [$Å^3$]	1965.8(2)
Z	4
ρ_{calc} [gcm^{-3}]	2.351
μ [mm^{-1}]	6.275
F(000)	1308
Crystal size [mm^3]	0.041×0.087×0.135
Crystal colour	clear dark red
Crystal shape	block
Radiation	MoK_α (λ=0.71073 Å)
2θ range [°]	4.76 to 50.83 (0.83 Å)
Index ranges	−11 ≤ h ≤ 11<br>−14 ≤ k ≤ 14<br>−21 ≤ l ≤ 19
Reflections collected	45260
Independent reflections	1810<br>R_{int} = 0.0665<br>R_{sigma} = 0.0151
Completeness to θ = 25.242°	99.8 %
Data / Restraints / Parameters	1810 / 0 / 156
Absorption correction T_{min}/T_{max} (method)	0.6395 / 0.7452 (multi-scan)
Goodness-of-fit on F^2	1.138
Final *R* indexes [$I \geq 2\sigma(I)$]	R_1 = 0.0257<br>wR_2 = 0.0576
Final *R* indexes [all data]	R_1 = 0.0272<br>wR_2 = 0.0583
Largest peak/hole [$eÅ^{-3}$]	0.78/−0.46

	**9Cl6F·**
CCDC number	2440953
Empirical formula	$C_{19}Cl_9F_6$
Formula weight	661.24
Temperature [K]	107.30
Crystal system	monoclinic
Space group (number)	$C2/c$ (15)
a [Å]	11.6598(15)
b [Å]	11.5791(12)
c [Å]	16.862(2)
α [°]	90
β [°]	109.566(4)
γ [°]	90
Volume [$Å^3$]	2145.1(4)

Z	4
ρ_{calc} [gcm^{-3}]	2.047
μ [mm^{-1}]	1.236
F(000)	1284
Crystal size [mm^3]	0.05×0.1×0.4
Crystal colour	red
Crystal shape	block
Radiation	MoK_α (λ=0.71073 Å)
2θ range [°]	5.11 to 51.57 (0.82 Å)
Index ranges	$-14 \le h \le 14$<br>$-14 \le k \le 12$<br>$-20 \le l \le 20$
Reflections collected	27819
Independent reflections	2058<br>R_{int} = 0.0970<br>R_{sigma} = 0.0399
Completeness to θ = 25.242°	100.0 %
Data / Restraints / Parameters	2058 / 0 / 156
Absorption correction T_{min}/T_{max} (method)	0.6288 / 0.7453 (none)
Goodness-of-fit on F^2	1.080
Final *R* indexes [$I \ge 2\sigma(I)$]	R_1 = 0.0683<br>wR_2 = 0.1968
Final *R* indexes [all data]	R_1 = 0.0912<br>wR_2 = 0.2142
Largest peak/hole [eÅ^{-3}]	1.32/−0.94

	**(3Cl12F-O)$_2$**
CCDC number	2440951
Empirical formula	$C_{38}Cl_6F_{24}O_2$
Formula weight	1157.08
Temperature [K]	100.00
Crystal system	monoclinic
Space group (number)	$P2_1/n$ (14)
a [Å]	13.3938(5)
b [Å]	18.7268(7)
c [Å]	15.2900(6)
α [°]	90
β [°]	94.317(2)
γ [°]	90
Volume [Å^3]	3824.2(3)
Z	4
ρ_{calc} [gcm^{-3}]	2.010
μ [mm^{-1}]	0.607
F(000)	2248
Crystal size [mm^3]	0.087×0.102×0.138
Crystal colour	clear light colourless
Crystal shape	prism

Radiation	MoK_{α} (λ=0.71073 Å)
2θ range [°]	4.20 to 50.73 (0.83 Å)
Index ranges	−16 ≤ h ≤ 16<br>−22 ≤ k ≤ 22<br>−18 ≤ l ≤ 18
Reflections collected	48916
Independent reflections	7010<br>R_{int} = 0.0419<br>R_{sigma} = 0.0232
Completeness to θ = 25.242°	100.0 %
Data / Restraints / Parameters	7010 / 0 / 633
Absorption correction T_{min}/T_{max} (method)	0.6922 / 0.7452 (multi-scan)
Goodness-of-fit on F^2	1.043
Final *R* indexes [I≥2σ(I)]	R_1 = 0.0420<br>wR_2 = 0.1024
Final *R* indexes [all data]	R_1 = 0.0510<br>wR_2 = 0.1093
Largest peak/hole [eÅ^{-3}]	1.32/−1.04

	**(3Br12F-O)$_2$**
CCDC number	2440949
Empirical formula	$C_{38}Br_6F_{24}O_2$
Formula weight	1423.84
Temperature [K]	100.00
Crystal system	monoclinic
Space group (number)	$P2_1/c$ (14)
a [Å]	11.4055(10)
b [Å]	19.3955(16)
c [Å]	18.7897(16)
α [°]	90
β [°]	106.243(3)
γ [°]	90
Volume [Å^3]	3990.7(6)
Z	4
ρ_{calc} [gcm^{-3}]	2.370
μ [mm^{-1}]	6.188
F(000)	2680
Crystal size [mm^3]	0.041×0.087×0.135
Crystal colour	clear light colourless
Crystal shape	block
Radiation	MoK_{α} (λ=0.71073 Å)
2θ range [°]	4.20 to 51.45 (0.82 Å)
Index ranges	−13 ≤ h ≤ 13<br>−23 ≤ k ≤ 23<br>−22 ≤ l ≤ 22
Reflections collected	78128
Independent reflections	7591<br>R_{int} = 0.0896<br>R_{sigma} = 0.0390

Completeness to θ = 25.242°	100.0 %
Data / Restraints / Parameters	7591 / 0 / 631
Absorption correction T_{min}/T_{max} (method)	0.6240 / 0.7453 (multi-scan)
Goodness-of-fit on F^2	1.027
Final *R* indexes [$I \geq 2\sigma(I)$]	R_1 = 0.0372 wR_2 = 0.0830
Final *R* indexes [all data]	R_1 = 0.0564 wR_2 = 0.0918
Largest peak/hole [eÅ^{-3}]	0.91/−0.89

6. Quantum-chemical Calculations

**3Cl12F·**	**(3Cl12F-O)$_2$**
34	70
Energy = -3302.524855045	Energy = -6755.407478508
C -0.0006385 0.0008099 0.0008701	C -12.8576210 -0.6330394 1.7962664
C -0.0533737 1.3877290 -0.4280820	C -13.2006300 0.8122514 1.3878517
C -1.2067754 -0.6640991 0.4617362	C -14.1040094 -1.5349207 1.6580005
C 1.2595835 -0.7210965 -0.0319182	C -11.5750070 -1.1551350 1.1165211
C 2.1870938 -0.5637790 -1.0738802	C -10.8775676 -0.5048511 0.1010536
C 1.6327127 -1.6246908 0.9755118	C -10.9634715 -2.3092241 1.6273441
C 2.8280409 -2.3144435 0.9491626	C -9.7285877 -2.7508885 1.1980429
C 3.7256869 -2.1392054 -0.0987815	C -9.0399107 -2.0701993 0.2008591
C 3.3839082 -1.2504347 -1.1126472	C -9.6399950 -0.9505619 -0.3485325
C -2.1708537 -0.0109278 1.2462641	C -15.3209264 -1.1374264 2.2270039
C -3.3180795 -0.6382746 1.6883755	C -16.4429865 -1.9460449 2.2355376
C -3.5699012 -1.9676375 1.3656384	C -16.4274115 -3.1923495 1.6270427
C -2.6338399 -2.6416284 0.5883578	C -15.2598801 -3.5780162 0.9905403
C -1.4890280 -2.0046538 0.1537803	C -14.1368221 -2.7629149 0.9979400
C -1.1293927 1.9044425 -1.1669376	C -14.0474011 1.0784320 0.3127753
C -1.1835063 3.2208692 -1.5784371	C -14.4189937 2.3588142 -0.0499032
C -0.1552818 4.1047136 -1.2680919	C -13.9319916 3.4565990 0.6465514
C 0.9244110 3.6225810 -0.5356356	C -13.0495164 3.2207332 1.6882640
C 0.9691184 2.3031895 -0.1324359	C -12.6767131 1.9296974 2.0352112
Cl -4.9929460 -2.7526472 1.9100335	Cl -17.8114874 -4.2025995 1.6377644
F -2.8441400 -3.9126650 0.2399466	F -15.2009261 -4.7436424 0.3446608
F -0.6518129 -2.7045711 -0.6225948	F -13.0966267 -3.2193819 0.2895053
F -1.9766738 1.2581990 1.6275657	F -15.4714819 0.0712196 2.7795439
F -4.1808264 0.0409229 2.4469320	F -17.5501525 -1.5077867 2.8398772
F -2.1371743 1.1021129 -1.5332545	F -14.5087876 0.0761731 -0.4526593
F -2.2294585 3.6366999 -2.2953901	F -15.2395730 2.5289905 -1.0880200
Cl -0.2171798 5.7413330 -1.7736896	Cl -14.3884786 5.0528788 0.2142932
F 1.9242444 4.4405289 -0.2004256	F -12.5304233 4.2413805 2.3729592
F 2.0214995 1.9178925 0.6007844	F -11.7699649 1.8305661 3.0115912
F 1.9081816 0.2476831 -2.1023523	F -11.3547772 0.5848922 -0.5096136
F 4.2076166 -1.0658563 -2.1463286	F -9.0308437 -0.2700691 -1.3191339
F 0.8341391 -1.8149700 2.0335855	F -11.5847712 -3.0684184 2.5355421

F 3.1239532 -3.1438581 1.9519683	F -9.2089604 -3.8523135 1.7423963
Cl 5.2106927 -2.9942883 -0.1396303	Cl -7.5075400 -2.6129784 -0.3413122
	O -12.3486175 -0.6575883 3.1515309
	O -13.4261314 -0.6907357 4.1231155
	C -12.9241695 -0.8945228 5.4696914
	C -12.5702416 0.4675397 6.0977593
	C -14.2131837 -1.4997005 6.0603762
	C -11.6928481 -1.8251698 5.4639781
	C -10.4713994 -1.3636118 4.9567701
	C -11.6782335 -3.1403420 5.9273698
	C -10.5670305 -3.9630724 5.8060359
	C -9.3946626 -3.5014554 5.2323602
	C -9.3616451 -2.1770847 4.8205274
	C -14.8961015 -1.0146926 7.1731119
	C -16.1385241 -1.5099137 7.5515974
	C -16.7568540 -2.5170520 6.8312136
	C -16.0813731 -3.0370689 5.7331619
	C -14.8417225 -2.5486844 5.3744715
	C -13.1281169 1.6663718 5.6555864
	C -12.7559333 2.8914108 6.1899361
	C -11.8387424 2.9724237 7.2254164
	C -11.3172680 1.7856514 7.7214129
	C -11.6899479 0.5726089 7.1736021
	Cl -18.2946661 -3.1201575 7.2875344
	F -16.6180123 -4.0297904 5.0215260
	F -14.2306329 -3.1617019 4.3557282
	F -14.3978029 -0.0486504 7.9521457
	F -16.7330284 -0.9885265 8.6245123
	F -14.0720380 1.7082662 4.7105200
	F -13.3097686 3.9996639 5.6949417
	Cl -11.3817613 4.4867372 7.8896415
	F -10.4599731 1.8014965 8.7437407
	F -11.1894009 -0.5288703 7.7558308
	F -10.3063591 -0.0868127 4.5946768
	F -8.2489632 -1.6677023 4.2862445
	F -12.7250845 -3.6867023 6.5581506
	F -10.6432095 -5.2131236 6.2652296

	Cl -8.0252653 -4.5176135 5.0634975
**3Br12F·**	**(3Br12F-O)$_2$**
34	70
Energy = -9644.161835000	Energy = -19438.69966115
C -0.0005611 0.0009365 0.0013762	C -12.8577815 -0.6335069 1.7964118
C -0.0535385 1.3880673 -0.4275962	C -13.1948638 0.8095900 1.3755214
C -1.2068263 -0.6642242 0.4622015	C -14.1113436 -1.5271667 1.6660601
C 1.2598628 -0.7210251 -0.0315400	C -11.5798195 -1.1706547 1.1196854
C 2.1862710 -0.5632037 -1.0746242	C -10.8708757 -0.5287898 0.1068310
C 1.6323037 -1.6240061 0.9769657	C -10.9841938 -2.3319668 1.6327091
C 2.8288975 -2.3145632 0.9508984	C -9.7558815 -2.7920269 1.2024623
C 3.7246154 -2.1383773 -0.0986770	C -9.0567925 -2.1206296 0.2079141
C 3.3844135 -1.2504866 -1.1139988	C -9.6386507 -0.9927297 -0.3408987
C -2.1695458 -0.0104252 1.2481180	C -15.3227901 -1.1182641 2.2383931
C -3.3182142 -0.6381337 1.6906396	C -16.4512004 -1.9188529 2.2530050
C -3.5689468 -1.9670606 1.3648132	C -16.4481582 -3.1644912 1.6457720
C -2.6348934 -2.6424263 0.5860804	C -15.2879738 -3.5600568 1.0043977
C -1.4886920 -2.0044978 0.1518640	C -14.1574099 -2.7547297 1.0059859
C -1.1301251 1.9032207 -1.1670692	C -14.0308618 1.0660938 0.2898475
C -1.1844397 3.2207343 -1.5800998	C -14.4030381 2.3434451 -0.0840725
C -0.1551461 4.1031253 -1.2686063	C -13.9287567 3.4456545 0.6118459
C 0.9250677 3.6236418 -0.5349887	C -13.0570390 3.2199815 1.6632057
C 0.9693661 2.3029554 -0.1308919	C -12.6822591 1.9323113 2.0225993
Br -5.1278278 -2.8267848 1.9604728	Br -17.9739524 -4.2585516 1.6639333
F -2.8409419 -3.9109476 0.2345844	F -15.2360876 -4.7258574 0.3569763
F -0.6506478 -2.7014326 -0.6249000	F -13.1238450 -3.2187217 0.2924622
F -1.9723394 1.2572993 1.6294242	F -15.4614616 0.0937974 2.7866896
F -4.1763121 0.0437699 2.4480671	F -17.5497673 -1.4654483 2.8630129
F -2.1359143 1.0990908 -1.5318606	F -14.4791483 0.0559954 -0.4735525
F -2.2307527 3.6309997 -2.2955288	F -15.2136996 2.4987649 -1.1328940
Br -0.2221115 5.8951547 -1.8242165	Br -14.4315971 5.1898151 0.1241897
F 1.9257027 4.4364046 -0.1985284	F -12.5466924 4.2433266 2.3514996
F 2.0194566 1.9160151 0.6032090	F -11.7842249 1.8418971 3.0078300
F 1.9047245 0.2477467 -2.1016026	F -11.3311878 0.5691861 -0.5024867
F 4.2034953 -1.0626696 -2.1478598	F -9.0220900 -0.3153319 -1.3097675
F 0.8327921 -1.8120620 2.0337444	F -11.6164554 -3.0788053 2.5437116
F 3.1198800 -3.1416013 1.9541139	F -9.2573344 -3.9019461 1.7501183

Br 5.3509277 -3.0752543 -0.1439840	Br -7.3847032 -2.7384818 -0.3824741<br>O -12.3470057 -0.6493381 3.1509128<br>O -13.4227274 -0.6779186 4.1251350<br>C -12.9211196 -0.8851381 5.4713117<br>C -12.5712638 0.4754353 6.1046427<br>C -14.2080106 -1.4980096 6.0584059<br>C -11.6851192 -1.8096380 5.4642216<br>C -10.4679681 -1.3415295 4.9531121<br>C -11.6606755 -3.1224227 5.9336813<br>C -10.5436254 -3.9378990 5.8122281<br>C -9.3772262 -3.4710534 5.2333445<br>C -9.3535888 -2.1493770 4.8163122<br>C -14.9022257 -1.0125874 7.1639149<br>C -16.1422996 -1.5186015 7.5380684<br>C -16.7453240 -2.5355647 6.8210085<br>C -16.0593221 -3.0555477 5.7310373<br>C -14.8234954 -2.5554311 5.3738694<br>C -13.1222247 1.6765434 5.6601763<br>C -12.7484334 2.8994163 6.1995670<br>C -11.8377043 2.9751403 7.2395409<br>C -11.3246929 1.7871960 7.7381560<br>C -11.6978093 0.5756230 7.1864037<br>Br -18.4271811 -3.2095224 7.3143232<br>F -16.5772603 -4.0584631 5.0192191<br>F -14.2019827 -3.1636385 4.3584362<br>F -14.4179321 -0.0360720 7.9390269<br>F -16.7440503 -0.9919328 8.6050372<br>F -14.0617779 1.7222395 4.7109209<br>F -13.2979344 4.0077155 5.6983703<br>Br -11.3339775 4.6316987 7.9706969<br>F -10.4730665 1.7928695 8.7659711<br>F -11.2059625 -0.5295549 7.7696213<br>F -10.3121503 -0.0639092 4.5894276<br>F -8.2479100 -1.6291609 4.2766483<br>F -12.7025869 -3.6715130 6.5706083<br>F -10.6158172 -5.1861218 6.2787028<br>Br -7.8695617 -4.5750914 5.0497251

9Cl6F·			
34			
Energy = -5464.438801652			
C	0.0006712	0.0001511	-0.0001690
C	-0.0531964	1.3947037	-0.4322456
C	-1.2127516	-0.6674863	0.4642280
C	1.2674614	-0.7263466	-0.0324617
C	2.1021887	-0.7004021	-1.1682147
C	1.7093269	-1.4851698	1.0704547
C	2.9101519	-2.1745450	1.0267000
C	3.7265409	-2.1387858	-0.0962082
C	3.3032119	-1.3905573	-1.1868783
C	-2.0712279	-0.0634542	1.4052090
C	-3.2218295	-0.7039181	1.8353413
C	-3.5689503	-1.9639517	1.3658706
C	-2.7280423	-2.5703308	0.4418796
C	-1.5771150	-1.9445841	-0.0086146
C	-1.0421467	1.8503076	-1.3276046
C	-1.0867672	3.1761509	-1.7268457
C	-0.1589242	4.1028181	-1.2695177
C	0.8221989	3.6639536	-0.3899600
C	0.8811370	2.3445308	0.0282912
Cl	-4.9948915	-2.7484053	1.9114027
F	-3.0616525	-3.7689057	-0.0361257
Cl	-0.6839418	-2.7499306	-1.2519324
Cl	-1.6856402	1.4439148	2.1611212
F	-4.0061614	-0.1206943	2.7413714
Cl	-2.1752852	0.7719261	-2.0659899
F	-2.0186925	3.5772443	-2.5910940
Cl	-0.2229406	5.7415788	-1.7762938
F	1.7035333	4.5476035	0.0776174
Cl	2.0722721	1.9535707	1.2201837
Cl	1.6297802	0.0876364	-2.6338996
F	4.0642759	-1.3674819	-2.2807922
Cl	0.8477082	-1.5085067	2.5702161
F	3.3154782	-2.8684411	2.0899064
Cl	5.2142197	-2.9941927	-0.1349458

Citations

[1] S. Stoll, A. Schweiger, EasySpin, a comprehensive software package for spectral simulation and analysis in EPR, *J. Magn. Reson.* **2006**, *178(1)*, 42.

[2] G. M. Sheldrick, A short history ofSHELX, *Acta Cryst.* **2008**, *A64*, 112.

[3] G. M. Sheldrick, Structure determination revisited, *Acta Cryst.* **2015**, *A71*, S9.

[4] O. V. Dolomanov, L. J. Bourhis, R. J. Gildea, J. A. K. Howard,H. Puschmann, OLEX2: a complete structure solution, refinement and analysis program, *J. Appl. Crystallogr.* **2009**, *42*, 339.

[5] TURBOMOLE GmbH, TURBOMOLE V7.3., a) development of University of Karlsruhe and Forschungszentrum Karlsruhe, **2018**.

[6] S. Grimme, J. Antony, S. Ehrlich, S. Krieg, A consistent and accurateab initioparametrization of density functional dispersion correction (DFT-D) for the 94 elements H-Pu, *J. Chem. Phys.* **2010**, *132*, 154104.

[7] S. Grimme, S. Ehrlich, L. Goerigk, Effect of the damping function in dispersion corrected density functional theory, *J. Comput. Chem.* **2011**, *32*, 1456.

[8] M. Sierka, A. Hogekamp, R. Ahlrichs, Fast evaluation of the Coulomb potential for electron densities using multipole accelerated resolution of identity approximation, *J. Chem. Phys.* **2003**, *118*, 9136.

[9] F. Neese, Software update: The ORCA program system—Version 5.0, *WIRES Comput. Molec. Sci.* **2022**, *12*, e1606.

[10] R. Krishnan, J. S. Binkley, R. Seeger, J. A. Pople, Self-consistent molecular orbital methods. XX. A basis set for correlated wave functions, *J. Chem. Phys.* **1980**, *72*, 650.

[11] A. D. McLean, G. S. Chandler, Contracted Gaussian basis sets for molecular calculations. I. Second row atoms, Z=11–18, *J. Chem. Phys.* **1980**, *72*, 5639.

[12] J.-P. Blaudeau, M. P. McGrath, L. A. Curtiss, L. Radom, Extension of Gaussian-2 (G2) theory to molecules containing third-row atoms K and Ca, *J. Chem. Phys.* **1997**, *107*, 5016.

[13] L. A. Curtiss, M. P. McGrath, J.-P. Blandeau, N. E. Davis, R. C. Binning, L. Radom, Extension of Gaussian-2 theory to molecules containing third-row atoms Ga–Kr, *J. Chem. Phys.* **1995**, *103*, 6104.

[14] T. Clark, J. Chandrasekhar, P. v. R. Schleyer, Efficient diffuse function-augmented basis sets for anion calculations. III. The 3-21+G basis set for first-row elements, Li–F, *J. Comp. Chem.* **1983**, *4*, 294.

[15] M. J. Frisch, J. A. Pople, J. S. Binkley, Self-consistent molecular orbital methods 25. Supplementary functions for Gaussian basis sets, *J. Chem. Phys.* **1984**, *80*, 3265.

[16] B. de Souza, G. Farias, F. Neese, R. Izsák, Predicting Phosphorescence Rates of Light Organic Molecules Using Time-Dependent Density Functional Theory and the Path Integral Approach to Dynamics, *J. Chem. Theory Comput.* **2019**, *15*, 1896.

[17] B. de Souza, F. Neese, R. Izsak, On the theoretical prediction of fluorescence rates from first principles using the path integral approach, *J. Chem. Phys.* **2018**, *148*, 034104.

[18] T. Behnke, C. Würth, K. Hoffmann, M. Hübner, U. Panne, U. Resch-Genger, Encapsulation of Hydrophobic Dyes in Polystyrene Micro- and Nanoparticles via Swelling Procedures, *J. Fluoresc.* **2011**, *21*, 937.

[19] T. Sato, Y. Hamada, M. Sumikawa, S. Araki, H. Yamamoto, Solubility of Oxygen in Organic Solvents and Calculation of the Hansen Solubility Parameters of Oxygen, *Ind. Eng. Chem. Res.* **2014**, *53*, *49*, 19331.
